# Psychometric evaluation of the professional forensic stigma scale

**DOI:** 10.3389/fpsyt.2026.1806480

**Published:** 2026-07-08

**Authors:** Ellen Vorstenbosch, Yvonne H. A. Bouman, Gemma Escuder-Romeva, Carolina Fellinghauer, Erik Bulten, Josep Maria Haro

**Affiliations:** 1 Research, Teaching and Innovation Unit, Parc Sanitari Sant Joan de Déu, Sant Boi de Llobregat, Spain; 2 Department of Medicine and Translational Research, University of Barcelona, Barcelona, Spain; 3 Swiss Paraplegic Research, Nottwil, Switzerland; 4 Centre for Biomedical Research on Mental Health (CIBERSAM), Madrid, Spain; 5 Department of Research, Transfore Forensic Mental Health, Deventer, Netherlands; 6 Penitentiary Psychiatric Hospitalization Unit of Catalonia, Parc Sanitari Sant Joan de Déu, Sant Esteve Sesrovires, Spain; 7 Research Group on Etiopathogenesis and Treatment of Severe Mental Disorders (MERITT) Group, Sant Joan de Déu Research Institute, Barcelona, Spain; 8 Division Diagnostics Research and Education, Forensic Psychiatric Centre Pompestichting, Nijmegen, Netherlands; 9 Behavioral Science Institute, Radboud University Nijmegen, Nijmegen, Netherlands

**Keywords:** forensic service users, mental healthcare professionals, professional stigma, psychometrics, Rasch analysis, scale development

## Abstract

**Background:**

Professional stigma toward forensic mental healthcare service users may compromise care quality and negatively affect treatment and rehabilitation outcomes. Reliable role- and context-specific instruments are needed to assess professional stigma in settings where therapeutic and security responsibilities intersect. This study examined the psychometric properties of the newly developed PROfessional Forensic Stigma (PROFS) scale, a multidimensional measure assessing stereotypes, prejudices, and discriminatory behaviors among professionals working with forensic populations.

**Methods:**

In this cross-sectional study, 219 mental healthcare professionals from forensic and community services in Spain (n = 79), Belgium (n = 83), and the Netherlands (n = 57) completed the initial PROFS (36 items; 7-point Likert scale), and measures of negative stereotypes (ATMIO), affective reactions (APDQ), social distance (SDS), empathy (IRI), and social desirability (BIDR-16). Psychometric evaluation used classical test theory and Rasch analysis (Partial Credit Model). Item functioning, dimensionality, reliability, and construct validity were assessed. Convergent and divergent validity were examined using Spearman correlation coefficients.

**Results:**

The initial PROFS demonstrated a three-domain structure (Stereotypes, Prejudice, Discrimination). Rasch-based item refinement and correction of disordered response thresholds resulted in a final 30-item version with a 5-point response format. The refined PROFS showed acceptable to good reliability (total scale PSI = 0.86–0.90; subscale PSI = 0.67–0.84). Convergent validity was supported by moderate to strong correlations between Stereotypes and negative stereotypes (ρ = 0.58), Prejudice and affective reactions (ρ = 0.40–0.55), and Discrimination and social distance (ρ = 0.36; all p < 0.01). Weak associations with empathy and social desirability supported divergent validity. Floor effects were observed for Discrimination items.

**Conclusions:**

The PROFS shows satisfactory psychometric performance and provides evidence for its reliability and construct validity as a measure of professional stigma among mental health care professionals working with forensic service users. The scale may support research and evaluation of stigma-related outcomes and can be used to identify professional stigma in teams or services and to inform the development and evaluation of stigma-reduction initiatives, with the ultimate aim of improving care and rehabilitation prospects for forensic service users.

## Introduction

Stigma is the social process of devaluating, discrediting and/or excluding individuals based on group association (1, 2). It emerges from the human tendency to categorize people, whereby labelling individuals as deviant reinforces a division between the ingroup (“us”) and the outgroup (“them”) (3). The stigma (i.e., the mark) can refer to a current, former, or even imagined condition, and may intersect with multiple stigmatized identities. The latter is the case for forensic service users, where dual labels of “mentally ill” and “offender” may further amplify their outgroup status (4–6). Beyond labelling, stigma unfolds through three interconnected mechanisms: stereotypes, prejudices and discrimination, representing the cognitive, emotional and behavioral components, respectively (7, 8). Stereotypes refer to beliefs and assumptions about forensic service users as a group (e.g. that they are dangerous or unpredictable), prejudice reflects negative emotional reactions linked to these beliefs (e.g. fear or anger), and discrimination captures behavioral tendencies such as avoidance and segregation (9, 10). Importantly, prejudice involves an active (cognitively and affectively) evaluative response, resulting in a negative emotional reaction. This means that people can be aware of stereotypes but not endorse them, and likewise, they may hold prejudices without necessarily engaging in discriminatory behavior. Stigma further requires power imbalances (1), where the dominant group possesses the social or institutional authority to translate labels into status loss and real disadvantages for the stigmatized group. In mental healthcare (MHC) settings, these dynamics underpin professional stigma, whereby professionals as members of the dominant group may hold and enact negative beliefs, emotions, and behaviors towards the people they are meant to help (11).

Although professional stigma has been studied in general MHC, these insights are seldom extended to forensic services. In general MHC, professionals report beliefs about dangerousness, unpredictability and personal weakness, alongside recovery pessimism and a desire for social distance (12–15). Although these findings concern non-forensic service users (i.e., individuals with mental illness only), such attitudes have been associated with reduced quality of care (14), as well as poorer treatment engagement and outcomes (16–18). Conversely, in prison contexts, more rehabilitative and supportive attitudes among professionals have been linked to less punitive responses and better support for efforts to change offending behavior (19–21). Despite this, empirical knowledge on the attitudes of MHC professionals towards forensic service users remains limited. Research shows that stigma among professionals intensifies when mental illness and offending history co-occur (22), and that forensic service users are perceived as more dangerous than those with only one of these characteristics (6). Other studies among nursing professionals in low- and medium-secure settings have reported negative attitudes, including pity, danger, coercion, segregation and avoidance (23). Comparative work further suggests that forensic MHC nurses demonstrated significantly more positive attitudes than general inpatient nurses, including lower preferred social distance and higher trust, willingness to provide care, and perceived ability to manage threat (24).

Existing instruments, however, typically measure public stigma or general professional attitudes (25, 26), and fail to capture the specific dynamics of relationships between professionals and forensic service users. They overlook the heterogeneity of the forensic population (e.g., diversity in diagnosis and offending histories), and the context-specific range of stigma expressions from subtle, everyday slights to overtly discriminatory practices (27). Moreover, forensic service users receive care in a range of forensic and community MHC settings, where professionals must constantly balance therapeutic care with custodial and risk management responsibilities (28–30). Yet, existing instruments do not account for these dual imperatives. In such settings, professionals may experience ambivalent emotions such as compassion, frustration, fear and anger (31, 32). These emotional responses occur within a climate of pervasive concern about risk and safety, which may heighten danger appraisals and reinforce related stereotypes (33), which in turn are associated with greater support for restrictive and discriminatory behaviors such as segregation, avoidance, coercion, and reduced intentions to help (34, 35). In addition, professionals may question forensic service users’ capacity for change (36). Such beliefs about limited competence and recovery pessimism may further foster paternalistic care practices (37), whereby professionals make decisions on behalf of service users and restrict opportunities for shared decision-making (38). Professionals’ (unintentional) beliefs and biases thus shape how forensic service users are understood and treated, and existing power imbalances within MHC settings make professional stigma particularly consequential for assessment, treatment, and rehabilitation. Because these processes influence everyday practice, there is a clear need for targeted assessment instruments and a more nuanced understanding of professional stigma in services that provide treatment for forensic service users.

Therefore, we developed the PROfessional Forensic Stigma scale (PROFS), building on mental illness stigma framework, which distinguish stereotypes, prejudice and discrimination as inter−related cognitive, affective and behavioral mechanisms of stigma (39), and on recent conceptual work that identified the key aspects and behavioral manifestations of professional stigma towards forensic service users (9). In this conceptual work, stereotypes were found to encompass attributions such as dangerousness, limited capacity to change or recover, and lack of competence; prejudices to include recovery pessimism, perceived dangerousness, and devaluation of the individual on the basis of their offenses; and discrimination to capture how professionals restrict service users’ opportunities or rights in everyday care (e.g., limiting access to leave, therapy or rehabilitative activities) and how they interact with them interpersonally (e.g., dismissive reactions, avoidance, or intrusive behaviors). These theoretical mechanisms and empirically derived domains guided item development, such that items were generated to represent each manifestation of professional stigma in everyday practice. The PROFS, therefore, uniquely operationalizes stereotypes, prejudice, and discrimination within the forensic service user–professional relationship, providing an instrument to identify and quantify professional stigma and to inform efforts to mitigate its impact on care and rehabilitation.

The primary aim of the current study is to examine the psychometric properties of the PROFS, including its internal structure, item and scale functioning, reliability, and convergent and divergent validity with relevant constructs (e.g., negative stereotypes, affective responses, and desired social distance, empathy, and social desirability). A secondary aim is to refine the PROFS into a psychometrically robust and practically applicable instrument (e.g. through item reduction and optimization of item and scale functioning) for use in future research and daily practice.

## Materials and methods

### Scale development and conceptualization

The PROFS was developed in several consecutive phases, following contemporary best−practice recommendations for scale construction and validation (40–43). First, a targeted literature review was conducted to identify potential aspects of professional stigma towards forensic service users, as well as existing instruments assessing stigma related to mental illness, (a history of) criminal offending, or both. All identified aspects (subscales) and items of these instruments were rated for relevance on a 7-point Likert scale by three independent researchers, and items with mean scores above 5.33 were selected for a Delphi study (44). In this second phase, 98 international experts (i.e., researchers on stigma or forensic MHC, professionals from forensic and community MHC, and forensic service users), evaluated the relevance of 85 pre-selected items on a 7-point Likert scale and proposed eight additional items reflecting new unique topics. After three iterative rounds, 26 items were retained, reflecting three stigma mechanisms: stereotypes (i.e., dangerousness, (in)ability to change, and lack of competence), prejudices (i.e., fear and recovery pessimism), and discrimination (i.e., denying rights and imposing restrictions). To deepen the understanding of professional stigma in daily practice, the third phase involved a focus group with seven forensic service users. Three major themes and eight concepts emerged: prejudicial assumptions and responses (i.e., recovery pessimism and dismissal of ambitions, perceived dangerousness and fear, and devaluation through offenses), discriminatory interactions and biases (i.e., differential treatment and restrictions, dismissive reactions and avoidance, and intrusive behaviors and threats), and ambiguity and anticipated stigma (i.e., ambiguity in detecting stigma, and anticipated stigma) (9).

For the fourth phase, the three stigma mechanisms were used to define the underlying constructs: *stereotypes* – cognitive component (i.e., generalized beliefs about forensic service users), prejudices – emotional component, (i.e., feelings or emotional reactions towards forensic service users), and discrimination – behavioral component, (i.e., actions or decisions involving forensic service users). To avoid response fatigue and an overly negative questionnaire, several positive, recovery-oriented items were added. Preliminary items were generated/created by the first author and workshopped with the other authors, to ensure coverage of the constructs and identified concepts. For each concept, items were intentionally written to reflect an increasing degree of stigma (i.e. ranging from mild to more severe expressions), so that variation in the intensity of stereotypical, emotional and behavioral responses could be captured. During the workshop, various items with different phrasings were discussed to align the constructs with practice in a manner that was both logically coherent and emotionally/behaviorally resonant. Subsequently, the items were forward and back translated into Dutch and Spanish. The first author translated the scale into Dutch and Spanish, and the second and third author translated it into English. Discrepancies were discussed, resulting in the first version of the PROFS. Seventeen MHC professionals then piloted the questionnaire in Spanish, English and Dutch, providing feedback on length, clarity and accessibility, response variability, simplicity, logic and construct precision. Based on the outcomes of the pilot study, no further adaptations were made.

### Study design and sample

The current study is observational, cross-sectional, involving MHC professionals engaged in the treatment and care of forensic service users in Spain, the Netherlands and Belgium. A non-probability sampling approach was employed to recruit participants across a range of service contexts, including forensic MHC hospitals, specialized wards within penitentiary settings, general MHC hospitals with forensic units, and community MHC services (e.g., outpatient or community-based rehabilitation). The minimal sample size was set at 180 participants, based on a 1:5 item-to-response ratio for scale validation (45). Eligible participants were professionals directly involved in the MHC of forensic service users (e.g., psychiatrists, psychologists, nurses, social pedagogues, social workers, occupational therapists) who were aged ≥ 18 years and had sufficient proficiency in Spanish or Dutch.

Within this non−probability framework, a combination of convenience and snowball sampling was employed to achieve a diverse sample of MHC professionals. Initial recruitment involved direct contact through the authors’ professional networks within forensic and community MHC services (2 in Spain, 3 in the Netherlands, and 2 in Belgium). Participants were invited to complete the study questionnaire and encouraged to share the invitation with eligible colleagues to maximize participation and sample size, and to capture a broad range of professional roles and service contexts.

### Instruments

#### Professional forensic stigma scale

The original version of the PROfessional Forensic Stigma scale (PROFS) consisted of 36 items across 3 domains: stereotypes, prejudices, and discrimination. Respondents are instructed to indicate to what extent they agree with each statement using a labelled 7-point Likert scale (0 = not at all, 1 = to a very small extent, 2 = to a small extent, 3 = to a moderate extent, 4 = to a large extent, 5 = to a very large extent, and 6 = completely). Positive and negative items are alternated to minimize acquiescence and avoid response fatigue; positive items (1, 2, 6, 13–15, 28–30) are reversely scored so that higher scores indicate higher levels of professional stigma. Item scores can be summed to obtain subscale and total PROFS scores.

#### Attitudes towards mentally ill offenders

The Negative Stereotypes subscale of the ATMIO (46) measures attitudes towards forensic service users (i.e., mentally ill offenders) from a public perspective. This subscale comprises 10 items scored on a 6-point Likert scale (0= strongly disagree, 5= strongly agree); higher scores reflect more endorsement of negative stereotypes. The ATMIO has demonstrated satisfactory psychometric properties (46, 47), with Cronbach’s α for Negative Stereotypes of 0.86 among future professionals working with forensic service users, i.e., social worker and criminal justice students (48).

#### Attitudes towards personality disorder questionnaire

The APDQ (49) has been used in secure MHC hospitals, prisons, and other MHC services. The APDQ measures affective reactions on a 6-point Likert scale (1 = never to 6 = always), with higher scores indicating more negative affect. Permission to adapt the APDQ for this study was granted (email correspondence, 14.04.2025). The term *personality disorder patients* was replaced with *forensic service users*. Three of the original subscales were used in this study: Security/Vulnerability (S/V; 11 items), Acceptance/Rejection (A/R; 4 items), and Purpose/Futility (P/F; 3 items), resulting in 18 items. Previous studies have reported acceptable test–retest reliability (r = 0.72–0.85).

#### Social distance scale

The SDS (50) has been used widely as a proxy indicator for discriminatory behavior in stigma research (51), and has seen many adaptations (52). The version of the SDS used in the present study has been specifically validated among MHC professionals (53), demonstrating excellent psychometric properties (Cronbach’s α = 0.90). This SDS consists of 14 items rated on a 5-point Likert scale (1 = definitely willing to 5 = definitely unwilling). Several items (2-7) are reverse scored. Higher total scores indicate a stronger desire for social distance, reflecting greater stigma.

#### Interpersonal reactivity index

The IRI (54) measures empathy in interpersonal contexts. We used the subscales Perspective Taking (PT; i.e., the tendency to adopt the viewpoint of others in everyday interactions), and Empathic Concern (EC; i.e., “other-oriented” feelings of sympathy and compassion for people facing difficulties), each comprising seven items rated on a 5-point Likert scale (0 = does not describe me well to 4 = describes me very well), with higher scores indicating greater dispositional empathy. Both subscales have shown solidinternal consistency, with Cronbach’s a ranging from 0.78 to 0.84 (55).

#### Balanced inventory of desirable responding short form

The BIDR-16 (56), a shortened version of the original 40-item BIDR (57), assesses two components of social desirability: Self-Deceptive Enhancement (SDE; i.e., unintentional exaggeration of positive traits) and Impression Management (IM; i.e., deliberate presentation of oneself in a socially favorable light). Each subscale consists of eight items rated on a 7-point Likert-scale (1 = strongly disagree to 7 = strongly agree), with higher scores indicating greater tendencies for social desirability. Internal consistency for the BIDR-16 has been found to be acceptable, with Cronbach’s a ranging from 0.63 to 0.82 for SDE and 0.66 to 0.73 for IM (56).

Additionally, the questionnaire included questions related to demographics, professional background, work setting, and the forensic service user population the participants are working with (e.g., the percentage of forensic service users in their caseload, diagnoses, type of offense; see **Table 1**).

**Table 1:** Participant characteristics by country (N = 219).

Characteristics	Spain	The Netherlands	Belgium	Total
	(n=79)	(n=57)	(n=83)	(N = 219)
*Demographic variables*				
Gender, n (%)				
Male	26 (32.9)	18 (31.6)	23 (27.7)	67 (30.6)
Female	53 (67.1)	38 (66.7)	60 (73.3)	151 (68.9)
Other ^a^	–	–	–	1 (0.5)
Age (years), M (SD)	44.0 (10.6)	41.6 (12.1)	39.5 (10.0)	41.7 (10.9)
*Professional background*				
Work setting, n (%)				
Forensic mental health hospital	2 (2.5)	34 (59.6)	25 (30.1)	61 (27.9)
Specialized forensic ward within prison	46 (58.2)	–	4 (4.8)	50 (22.8)
General mental health hospital with forensic wards	16 (20.3)	1 (1.8)	26 (31.3)	43 (19.6)
Community-based forensic service	2 (2.5)	22 (38.6)	20 (24.1)	44 (19.9)
Community-based general mental health	9 (11.4)	–	7 (8.4)	16 (7.3)
Other	4 (5.1)	–	1 (1.2)	5 (2.3)
Current professional role, n (%)				
Psychiatrist	11 (13.9)	2 (3.5)	2 (2.4)	15 (6.8)
Psychologist	18 (22.8)	20 (35.1)	13 (15.7)	51 (23.3)
Social educator/pedagogue	14 (17.7)	18 (31.6)	14 (16.9)	46 (21.0)
Other therapeutic professionals	8 (10.1)	7 (12.3)	15 (18.1)	30 (13.7)
Nursing staff	25 (31.6)	6 (10.5)	23 (27.7)	54 (24.7)
Other	3 (3.7)	4 (7.0)	16 (19.3)	23 (10.5)
Years of professional experience with forensic service users, M (SD)	11.3 (8.5)	11.6 (9.6)	11.0 (10.0)	11.2 (8.7)
Level of contact with forensic service users, n (%)				
Daily or almost daily	55 (69.6)	43 (75.4)	62 (74.7)	160 (73.1)
Several times a week	10 (12.7)	12 (21.1)	13 (15.7)	35 (16.0)
Once a week or less	14 (17.7)	2 (3.5)	8 (9.8)	24 (11.0)
*Characteristics of forensic service users treated by participants*				
Forensic service users in caseload, n (% valid) ^b^				
<40%	25 (31.6)	3 (5.3)	16 (19.3)	4 (20.1)
40-79%	14 (17.7)	18 (31.6)	9 (10.8)	41 (18.7)
≥80%	39 (49.4)	36 (63.2)	58 (69.9)	133 (60.7)
Mental health conditions ^c^				
Psychotic disorders	62 (78.5)	40 (70.2)	51 (61.4)	153 (69.9)
Personality disorders	48 (60.8)	57 (100.0)	68 (81.9)	173 (79.0)
Neurodevelopmental disorders	16 (20.3)	15 (26.3)	22 (26.5)	53 (24.2)
Mood disorders	26 (32.9)	19 (33.3)	21 (25.3)	66 (30.1)
Other	9 (11.4)	7 (12.3)	26 (31.3)	42 (19.2)
Offense types ^c^				
Violent offenses	71 (89.9)	57 (100.0)	67 (80.7)	195 (89.0)
Sexual offenses (adults)	20 (25.3)	30 (52.6)	36 (43.4)	86 (39.3)
Sexual offenses (minors)	16 (20.3)	43 (75.4)	38 (45.8)	97 (44.3)
Property offenses	43 (54.4)	17 (29.8)	16 (19.3)	76 (34.7)
Other	4 (5.1)	4 (7.0)	5 (6.0)	13 (5.9)

a To protect participant confidentiality, categories with very small cell sizes are not reported at the country level.

b Percentages are based on valid responses; missing values were in Spain (n=1, 1.3%).

c Multiple responses allowed; percentages may exceed 100%.

### Data collection

Data collection took place from June to November 2025 using an online questionnaire administered via Qualtrics (Qualtrics, Provo, UT). The questionnaire was available in Spanish and Dutch to accommodate participants in the respective countries. Participation was anonymous and voluntary, which is particularly recommended for stigma-related content as it can help to reduce socially desirable responding (58). Due to the open design, it was not possible to determine the response rate or verify whether participants completed the questionnaire more than once. Before accessing the questionnaire, participants were presented with an electronic informed consent form outlining the study objectives, eligibility criteria, and rights regarding participation. Only participants who provided explicit consent were able to proceed to the questionnaire. Participants could withdraw at any point without negative consequences. All data were treated confidentially and stored securely on access-restricted servers. Prior to the study, ethical approval was obtained from the ethics committee of Fundación Sant Joan de Déu (reference: C.I. PIC-55-25).

### Data analyses

Participants who provided no valid responses on any stigma-related item were classified as non-respondents (59). Cases with more than 20% missing responses on the PROFS (i.e. > 7 missing items on the 36-item scale) were excluded from PROFS-specific analyses. For all other analyses, PROFS scores were based on available items, whereas for the Rasch analyses remaining PROFS item-level missing values were imputed using a random-forest-based approach (60).

Demographic and work-related characteristics of the final sample were calculated using descriptive statistics. Continuous variables were described with means and standard deviations (SD). Categorical variables were reported using absolute and relative frequencies. Current professional role was recorded using predefined categories; for analysis, these roles were collapsed into five groups: psychiatrists, psychologists, social educators/pedagogues, nursing staff (nurses and nurse specialists), other therapeutic professionals (e.g., occupational, exercise/sports, drama), and others (e.g., peer support workers, team coach, unit manager, or program coordinator). For patient-population variables with multiple response options (e.g., diagnoses, offense history), answers were recoded into separate yes/no indicators and reported as counts and percentages, acknowledging that percentages could exceed 100%. Differences in participant characteristics between countries were examined using chi-square tests for categorical variables and ANOVA for continuous variables (two-sided significance level *p* <.05, without adjustment for multiple comparisons, as these analyses were considered descriptive).

Descriptive item analyses, reflecting classical test theory (CTT) statistics (number of missing values, means, standard deviations, skewness, kurtosis, range, floor and ceiling effects, and corrected item–total correlations), were conducted for all PROFS items. Floor and ceiling effects were defined as more than 15% endorsed the lowest or highest possible score, respectively (61). Corrected item-total correlations below. 30 were considered suboptimal and used as an indicator for potential item removal (40).

To examine the higher-order dimensional structure of the PROFS, exploratory factor analysis (EFA) was conducted using principal axis factoring with oblimin rotation to accommodate potentially correlated dimensions. Sampling adequacy was verified with the Kaiser-Meyer-Olkin (KMO) index and Bartlett’s test of sphericity; with KMO values > 0.70 considered acceptable (62). The number of factors was guided by eigenvalues greater than one, inspection of the scree plot, and substantive interpretability of factor content.

Rasch modeling, an item response theory approach, was used to examine the measurement properties of the PROFS scale. When the data fit the model, interval-scaled measures can be derived from responses originally collected on an ordinal scale. In these models, the probability of endorsing a response option is modelled as a function of the item location (difficulty) and the person parameter, here the respondent’s position on the latent professional stigma continuum. Respondents with higher stigma levels, relative to an item’s location, therefore, have a higher probability of choosing more stigmatizing response categories. The Partial Credit Model (PCM), an extension of the classical Rasch model for dichotomous data that can handle ordinal-scaled response options (63), was applied to all PROFS items. Key measurement assumptions were evaluated, including item fit, ordered response thresholds, absence of local item dependence (LID), unidimensionality, differential item functioning (DIF) across gender, age group and country scores, and targeting (match between item locations and respondents’ attitudes) (64). Item fit was evaluated with infit and outfit mean square statistics (values > 1.2 indicating underfit and values < 0.5 considered overfit but not harmful for measurement) (65), LID was examined via correlations among standardized residuals (item pairs ≥ 0.30 above the average residual correlation flagged), and unidimensionality was assessed by principal component analysis (PCA) of residuals (66), with a first residual eigenvalue > 2.0 taken as evidence of multidimensionality. Differential item functioning (DIF) was examined using the partial gamma approach for polytomous Rasch items across gender, age group, and country, and was considered problematic when subgroup differences in item difficulty were statistically significant with partial gamma coefficients ≥ 0.20 (67, 68). Targeting was evaluated by inspecting the match between item locations and respondents’ stigma levels on the latent continuum, using person–item maps and summary statistics to assess whether the items adequately covered the range of attitudes in the sample. Rasch analyses were used iteratively to guide item reduction and refinement of the PROFS. Response categories were collapsed where necessary to obtain ordered thresholds. After each run, items were reviewed on model fit, LID, DIF, and CTT statistics, and were retained or removed based on these indices and the conceptual relevance of their content. The Rasch analyses and their reporting were informed by current recommendations and guidelines for Rasch measurement (69).

For PROFS total and subscale scores, we used non-parametric descriptive statistics and report medians and interquartile ranges (IQR) for the total sample and by country.

Internal consistency and reliability of the final PROFS subscales were evaluated within the Rasch framework using the Person Separation Index (PSI) and Cronbach’s alpha. The PSI can be interpreted similarly to Cronbach’s alpha; values of 0.70 or higher were considered acceptable, with values ≥ 0.80 indicating good and ≥ 0.90 excellent internal consistency (70). Cronbach’s alpha was additionally calculated for the total PROFS as well as each subscale separately by country.

For PROFS total and subscale scores, raw summed scores were calculated, and medians and interquartile ranges (IQR) were computed for the total sample and by country. At the item level, response frequencies for each response category were calculated for the total sample and separately by country.

To examine convergent and divergent construct validity of the PROFS subscales, Spearman’s rank correlations were computed between PROFS subscale scores and the other measures (i.e., ATMIO Negative Stereotypes, APDQ and its subscales, SDS, IRI and its subscales, and BIDR-16 and its subscales). We expected moderate to strong positive correlations between PROFS Stereotypes and ATMIO Negative Stereotypes, between PROFS Prejudice and the APDQ subscales, and between PROFS Discrimination and the SDS. For divergent validity, Spearman’s rank correlations were computed for the PROFS subscale scores with the IRI and the BIDR-16. The PROFS subscales were expected to show only weak or negligible correlations with the IRI and BIDR-16 Total and their subscales. Correlations of. 10 – .29 were considered weak, .30 – .49 moderate and ≥ .50 were defined as strong (71).

Descriptive analyses for participant characteristics, item-level statistics, and Spearman’s rank correlations were conducted using IBM SPSS Statistics version 28.0. Descriptive analyses for PROFS total and subscale raw summed scores (medians and interquartile ranges, IQR) and item-level response frequencies, as well as analyses of the PROFS scales within the Rasch framework, were performed using R software (version 4.5.1), using the packages missRanger for missing-value imputation (72), eRm for Rasch-based psychometric analyses (73), and iarm for differential item functioning (DIF) analyses (74).

## Results

Overall, 281 persons opened the questionnaire and 262 consented to participate (93.2%). Of those, 33 (12.6%) did not respond to any stigma-related item and were considered non-respondents for the stigma scales. Of the remaining 229 participants, 221 (84.4%) met the ≥ 80% completion criterion for the PROFS (i.e., ≤ 7 missing items). Two participants reported never having contact with forensic service users and were therefore excluded from analyses, resulting in a final sample of N = 219 participants (**Table 1**). Most participants worked in Belgium (n=83; 37.9%) or Spain (n=79; 36.1%), followed by the Netherlands (n = 57; 26.0%).

Participants from the three countries differed significantly in age (*p* = 0.03), work setting (*p* <.001), and professional role (*p* <.001). Spanish MHC professionals were older than Belgian professionals (M = 44.0 vs. 39.5 years; SDs = 10.6 and 10.0, respectively), whereas Dutch professionals (M = 41.6 years, SD = 12.1) did not differ significantly from either group. Regarding work setting, Dutch professionals predominantly worked in forensic mental health hospitals (59.6%), Spanish professionals mainly in specialized forensic wards within prisons (58.2%), and Belgian professionals were more evenly distributed across forensic mental health hospitals (30.1%), general mental health hospitals with forensic wards (31.3%), and community-based forensic services (24.1%). In terms of professional role, Spanish participants were most often nursing staff (31.6%) and psychologists (22.8%), Dutch participants predominantly psychologists (35.1) and social educators/pedagogues (31.6%), and Belgian participants were mainly employed as nursing staff (27.7%), other psychosocial therapists (18.1%), and in other roles (19.3%).

Participants further differed significantly in the characteristics of the forensic service users they worked with, including the proportion of forensic service users in their caseload, the prevalence of personality disorder or other disorders (i.e., mainly substance abuse), as well as the histories of violent offenses, sexual offenses involving adults, sexual offenses involving minors, and property offenses (all *p* ≤.004).

**Figure 1:**
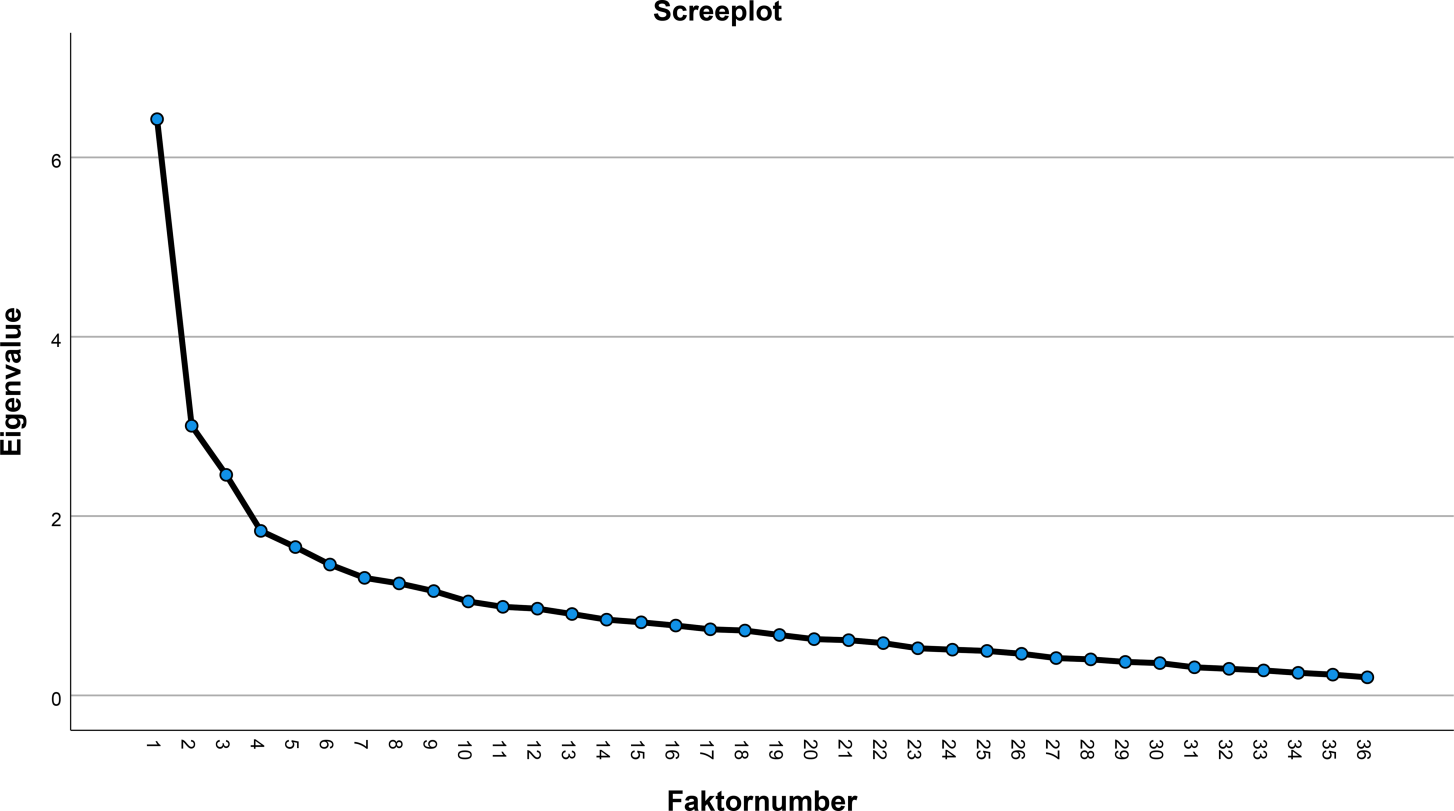
Scree plot of the exploratory factor analysis of the PROFS.

The 36−item scale yielded a participant−to−item ratio of 6:1. Sampling adequacy for factor analyses was supported by a KMO of 0.80 and a significant Bartlett’s test of sphericity (χ²(630) = 2719.2, *p* <.001). The exploratory factor analysis scree plot and eigenvalues > 1.0 for the first five factors indicated a multidimensional structure, with a clear elbow after the third–fourth factor where eigenvalues levelled off (**Figure 1**). Accordingly, the screen test rather than the Kaiser criterion was used to determine a three-factor solution consistent with the hypothesized PROFS domains (Stereotypes, Prejudice, Discrimination). Rasch analyses were subsequently conducted separately to assess whether items within each domain met unidimensional measurement requirements.

### Iterative process of item reduction

The Rasch analyses of all 36 items, using principal component analysis (PCA) of residuals, also indicated multidimensionality (first residual eigenvalue = 3.57), supporting the use of the PROFS domains (Stereotypes, Prejudice, Discrimination). Rasch analyses were conducted iteratively: first using the original 7-point response categories and then, after addressing disordered thresholds by collapsing adjacent categories, using a 5-category scale (1, 2-3, 4, 5-6, 7) for all PROFS items. Descriptive statistics and Rasch results for the original 7-point response format are provided in the (**Supplementary Tables 1**, **2**).

Item reduction was based on the Rasch models with collapsed categories, considering item fit, LID, DIF and CTT statistics for each PROFS subscale (**Supplementary Tables 3**, **4**). Item 6 (Stereotypes; outfit MSQ = 1.78, infit MSQ = 1.58) and item 16 (Prejudice; outfit MSQ = 1.63, infit MSQ = 1.50) showed clear misfit and very low corrected item–total correlations (CITC = -.006 and −.005, respectively) and were excluded. Item 29 (Discrimination) showed acceptable Rasch fit but a relatively low corrected item–total correlation (CITC = .173) and was also removed. In contrast, items 25 and 35 (Discrimination) showed acceptable Rasch fit and no significant DIF, although they had pronounced floor effect (83.1% and 82.6%, respectively); because removing these items did not improve overall model fit or reliability, they were retained. A subsequent Rasch analysis on the reduced item set identified additional misfit for item 12 (Stereotypes; outfit MSQ = 1.20) and item 30 (Discrimination; outfit MSQ = 1.35, infit MSQ = 1.30), which were therefore removed. Although item 15 (Prejudice) did not show notable misfit, it had a relatively low corrected item–total correlation (CITC = .183) and the first contrast in the residual PCA of the Prejudice subscale remained high (eigenvalue = 2.70); removing item 15 reduced this eigenvalue (2.19) and improved unidimensionality, so this item was excluded. In the final solution, item 13 (Prejudice) showed slight residual misfit (outfit MSQ = 1.40, infit MSQ = 1.31), but was conceptually relevant and its removal did not meaningfully improve overall fit or reliability; it was therefore retained.

After removing items 6, 12, 15, 16, 29 and 30, the Rasch models with collapsed categories showed satisfactory item fit across the PROFS subscales (**Supplementary Table 5**). Infit and outfit mean square values for 29 of the 30 remaining items fell within the predefined acceptable range. Disordered thresholds were observed for 4 items in Stereotypes, 3 in Prejudice, and 7 in Discrimination, indicating that respondents did not use the response categories in a strictly ordered manner for these items. Differential item functioning (DIF) analyses showed no evidence of non−invariance for any item across gender or age groups. One item showed non−invariance across countries: item 31 (Discrimination) demonstrated significant uniform DIF by country (γ_p_ = -0.45, Bonferroni−adjusted *p* <.001). Removing this item slightly reduced subscale reliability and did not improve global fit; given its conceptual importance and the modest magnitude of the DIF effect, this item was retained. Local item dependency was no longer observed (residual correlations < 0.30).

PCA of Rasch residuals indicated small residual dimensions for the Stereotypes and Discrimination subscales (first contrast eigenvalues = 1.93 and 1.52, respectively), whereas the Prejudice subscale showed a somewhat higher first contrast eigenvalue (2.19), suggesting a minor secondary component. For several items, particularly within the Discrimination subscale, pronounced floor effects remained, indicating that many professionals endorsed the least stigmatizing response categories. Across subscales, item–person targeting indicated that the PROFS items generally represented higher levels of stigma than those endorsed by respondents. For Stereotypes, mean item and mean person location were 2.74 and -1.12 logits, for Prejudice 1.87 and −1.67 logits, and for Discrimination 1.07 and −2.72 logits, respectively, suggesting suboptimal targeting at lower levels of professional stigma across all three subscales. Illustrative person–item maps for each subscale are provided in (**Supplementary Figures 1**–**3**).

### PROFS scores and item responses

**Table 2** presents descriptive statistics (median, IQR and observed range) for the PROFS total and subscale scores in the total sample and by country, as well as for the external measures included in the construct validity analyses. At the item level, most respondents selected the least stigmatizing response categories, particularly for Discrimination items. Item-level response frequencies for each response category in the total sample and by country are presented in **Table 3**. This table includes all 36 original PROFS items to allow inspection of response patterns for both retained and removed items.

**Table 2:** Descriptive statistics of PROFS total and subscale scores and external validity measures by country (N = 219).

Measure	Range ^a^	Spain (n=79)		The Netherlands (n=57)		Belgium (n=83)		Total (N = 219)	
		Median (IQR)	Min-Max ^b^	Median (IQR)	Min-Max ^b^	Median (IQR)	Min-Max ^b^	Median (IQR)	Min-Max ^b^
PROFS Total	0-120	30 (20.5)	4-59	32 (11)	13-55	28 (9.5)	14-56	30 (12.5)	4-59
Stereotypes	0-40	13 (7)	2-28	13 (6)	6-27	13 (6.5)	4-24	13 (7)	2-28
Prejudice	0-40	11 (7)	1-24	10 (4)	1-18	9 (3)	3-25	10 (5)	1-25
Discrimination	0-40	6 (6.5)	0-17	7 (6)	0-14	6 (3.5)	0-15	6 (5)	0-17
ATMIO NS	0-50	6 (9)	0-27	7 (8)	0-22	5 (7)	0-22	5 (8)	0-27
APDQ S/V	11-66	19 (8)	11-36	22 (8)	12-29	18 (6)	11-27	19 (8)	11-36
APDQ A/R	4-24	7 (3)	4-12	8 (2)	4-13	6 (3)	4-15	7 (3)	4-15
APDQ P/F	3-18	6 (3)	4-12	7 (2)	3-10	6 (3)	3-11	6 (3)	3-13
SDS	14-70	30 (12)	17-55	36 (11)	18-51	33 (10)	15-55	33 (11)	15-55
IRI EC	0-28	22 (7.5)	10-28	18 (5)	7-25	19 (5)	11-26	20 (6)	7-28
IRI PT	0-28	21 (6)	8-28	20 (4)	28-57	21 (5)	9-26	21 (5)	8-28
BIDR-16	16-112	73 (16.8)	29-101	71 (13)	45-89	71 (12.8)	50-100	72 (15)	29-101
BIDR-16-SDE	8-56	32 (10.8)	8-51	35 (9)	16-48	34.5 (9.8)	19-48	34 (9)	8-51
BIDR-16-IM	8-56	42 (13.8)	19-55	37 (8)	17-53	36 (8)	22-55	38 (11)	17-55

a Range, possible score range; ^b^Min-Max, observed minimum – maximum.

IQR, Interquartile range.

ATMIO NS, Negative Stereotypes subscale of the Attitudes towards Mentally Ill Offenders (ATMIO); APDQ S/V, Security/Vulnerability subscale of the Attitudes towards Personality Disorder Questionnaire (APDQ); APDQ A/R, Acceptance/Rejection subscale of the APDQ; APDQ P/F, Purpose/Futility subscale of the APDQ; SDS, Social Distance Scale; IRI EC, Empathic Concern subscale of the Interpersonal Reactivity Index (IRI); IRI PT, Perspective Taking subscale of the IRI; BIDR-16, Balanced Inventory of Desirable Responding–Short Form; BIDR-16 SDE, Self-Deceptive Enhancement subscale of the BIDR-16; BIDR-16 IM, Impression Management subscale of the BIDR-16.

Sample sizes vary slightly across measures due to missing data (range of n per country: Spain 74-79, Belgium 82-83).

**Table 3:** Item-level response frequencies for all evaluated PROFS items by response category, total sample and by country (collapsed 5-category format, 0–4 scoring) ^a.^.

Item Nr.	Item	Response option ^b,c^	Spain (n=79)	The Netherlands (n=57)	Belgium (n=83)	Total (N = 219)
			n (%)	n (%)	n (%)	n (%)
*Stereotypes*						
1	Most of these patients can learn to control their impulses *	Not at all	5 (6.3)	–	2 (2.4)	7 (3.2)
		To a small extent	31 (39.2)	25 (43.9)	36 (43.4)	92 (42)
		To a moderate extent	25 (31.6)	25 (43.9)	31 (37.3)	81 (37)
		To a large extent	18 (22.8)	7 (12.3)	14 (16.9)	39 (17.8)
		Completely	–	–	–	–
2	Most of these patients can make a positive contribution to society *	Not at all	5 (6.3)	1 (1.8)	5 (6)	11 (5)
		To a small extent	38 (48.1)	21 (36.8)	40 (48.2)	99 (45.2)
		To a moderate extent	16 (20.3)	24 (42.1)	25 (30.1)	65 (29.7)
		To a large extent	20 (25.3)	11 (19.3)	13 (15.7)	44 (20.1)
		Completely	–	–	–	–
3	Most of these patients lack a sense of right and wrong	Not at all	15 (19)	2 (3.5)	8 (9.6)	25 (11.4)
		To a small extent	42 (53.2)	29 (50.9)	48 (57.8)	119 (54.3)
		To a moderate extent	11 (13.9)	13 (22.8)	18 (21.7)	42 (19.2)
		To a large extent	11 (13.9)	13 (22.8)	9 (10.8)	33 (15.1)
		Completely	–	–	–	–
4	In general, these patients do not really want to change	Not at all	16 (20.3)	3 (5.3)	9 (10.8)	28 (12.8)
		To a small extent	44 (55.7)	38 (66.7)	57 (68.7)	139 (63.5)
		To a moderate extent	14 (17.7)	12 (21.1)	13 (15.7)	39 (17.8)
		To a large extent	5 (6.3)	4 (7)	4 (4.8)	13 (5.9)
		Completely	–	–	–	–
5	These patients use their mental disorder to avoid responsibility	Not at all	14 (17.7)	7 (12.3)	17 (20.5)	38 (17.4)
		To a small extent	36 (45.6)	38 (66.7)	55 (66.3)	129 (58.9)
		To a moderate extent	19 (24.1)	7 (12.3)	8 (9.6)	34 (15.5)
		To a large extent	10 (12.7)	5 (8.8)	3 (3.6)	18 (8.2)
		Completely	–	–	–	–
6	These patients are not to blame for their current situation *	Not at all	–	1 (1.8)	–	1 (0.5)
		To a small extent	22 (27.8)	4 (7)	8 (9.6)	34 (15.5)
		To a moderate extent	24 (30.4)	8 (14)	23 (27.7)	55 (25.1)
		To a large extent	29 (36.7)	41 (71.9)	45 (54.2)	115 (52.5)
		Completely	3 (3.8)	3 (5.3)	7 (8.4)	13 (5.9)
7	In general, these patients lack the capacity to change	Not at all	11 (13.9)	3 (5.3)	2 (2.4)	16 (7.3)
		To a small extent	42 (53.2)	34 (59.6)	47 (56.6)	123 (56.2)
		To a moderate extent	20 (25.3)	14 (24.6)	21 (25.3)	55 (25.1)
		To a large extent	6 (7.6)	6 (10.5)	13 (15.7)	25 (11.4)
		Completely	–	–	–	–
8	These patients manipulate to gain special treatment or privileges	Not at all	9 (11.4)	1 (1.8)	8 (9.6)	18 (8.2)
		To a small extent	36 (45.6)	24 (42.1)	50 (60.2)	110 (50.2)
		To a moderate extent	18 (22.8)	17 (29.8)	12 (14.5)	47 (21.5)
		To a large extent	15 (19)	15 (26.3)	13 (15.7)	43 (19.6)
		Completely	1 (1.3)	–	–	1 (0.5)
9	These patients suddenly become aggressive, without any apparent cause	Not at all	20 (25.3)	5 (8.8)	11 (13.3)	36 (16.4)
		To a small extent	46 (58.2)	40 (70.2)	64 (77.1)	150 (68.5)
		To a moderate extent	11 (13.9)	7 (12.3)	7 (8.4)	25 (11.4)
		To a large extent	2 (2.5)	5 (8.8)	1 (1.2)	8 (3.7)
		Completely	–	–	–	–
10	Most of these patients pose an inherent risk to others	Not at all	14 (17.7)	–	6 (7.2)	20 (9.1)
		To a small extent	45 (57)	30 (52.6)	51 (61.4)	126 (57.5)
		To a moderate extent	13 (16.5)	11 (19.3)	15 (18.1)	39 (17.8)
		To a large extent	7 (8.9)	16 (28.1)	10 (12)	33 (15.1)
		Completely	–	–	1 (1.2)	1 (0.5)
11	Most of these patients will reoffend sooner or later	Not at all	3 (3.8)	2 (3.5)	2 (2.4)	7 (3.2)
		To a small extent	37 (46.8)	42 (73.7)	45 (54.2)	124 (56.6)
		To a moderate extent	24 (30.4)	7 (12.3)	27 (32.5)	58 (26.5)
		To a large extent	14 (17.7)	6 (10.5)	8 (9.6)	28 (12.8)
		Completely	1 (1.3)	–	–	1 (0.5)
12	These patients will always depend on others	Not at all	8 (10.1)	2 (3.5)	3 (3.6)	13 (5.9)
		To a small extent	36 (45.6)	23 (40.4)	39 (47)	98 (44.7)
		To a moderate extent	20 (25.3)	13 (22.8)	23 (27.7)	56 (25.6)
		To a large extent	15 (19)	18 (31.6)	16 (19.3)	49 (22.4)
		Completely	–	1 (1.8)	2 (2.4)	3 (1.4)
*Prejudice*						
13	I feel compassion for these patients *	Not at all	3 (3.8)	4 (7)	4 (4.8)	11 (5)
		To a small extent	29 (36.7)	28 (49.1)	29 (34.9)	86 (39.3)
		To a moderate extent	25 (31.6)	20 (35.1)	20 (24.1)	65 (29.7)
		To a large extent	18 (22.8)	5 (8.8)	25 (30.1)	48 (21.9)
		Completely	4 (5.1)	–	5 (6)	9 (4.1)
14	I feel optimistic about the future of these patients *	Not at all	–	1 (1.8)	2 (2.4)	3 (1.4)
		To a small extent	24 (30.4)	16 (28.1)	26 (31.3)	66 (30.1)
		To a moderate extent	25 (31.6)	27 (47.4)	36 (43.4)	88 (40.2)
		To a large extent	28 (35.4)	13 (22.8)	19 (22.9)	60 (27.4)
		Completely	2 (2.5)	–	–	2 (0.9)
15	I feel protective of these patients *	Not at all	4 (5.1)	2 (3.5)	2 (2.4)	8 (3.7)
		To a small extent	24 (30.4)	22 (38.6)	27 (32.5)	73 (33.3)
		To a moderate extent	28 (35.4)	22 (38.6)	34 (41)	84 (38.4)
		To a large extent	17 (21.5)	10 (17.5)	20 (24.1)	47 (21.5)
		Completely	6 (7.6)	1 (1.8)	–	7 (3.2)
16	I feel pity for these patients	Not at all	16 (20.3)	5 (8.8)	10 (12)	31 (14.2)
		To a small extent	30 (38)	23 (40.4)	42 (50.6)	95 (43.4)
		To a moderate extent	21 (26.6)	16 (28.1)	14 (16.9)	51 (23.3)
		To a large extent	11 (13.9)	13 (22.8)	15 (18.1)	39 (17.8)
		Completely	–	–	2 (2.4)	2 (0.9)
17	I feel indifferent towards the problems of these patients	Not at all	53 (67.1)	27 (47.4)	50 (60.2)	130 (59.4)
		To a small extent	24 (30.4)	29 (50.9)	27 (32.5)	80 (36.5)
		To a moderate extent	1 (1.3)	1 (1.8)	5 (6)	7 (3.2)
		To a large extent	1 (1.3)	–	1 (1.2)	2 (0.9)
		Completely	–	–	–	–
18	I feel frustrated with the slow progress of these patients	Not at all	9 (11.4)	7 (12.3)	14 (16.9)	30 (13.7)
		To a small extent	28 (35.4)	40 (70.2)	48 (57.8)	116 (53)
		To a moderate extent	20 (25.3)	10 (17.5)	14 (16.9)	44 (20.1)
		To a large extent	16 (20.3)	–	6 (7.2)	22 (10)
		Completely	6 (7.6)	–	1 (1.2)	7 (3.2)
19	I feel overwhelmed when treating these patients	Not at all	13 (16.5)	14 (24.6)	31 (37.3)	58 (26.5)
		To a small extent	47 (59.5)	32 (56.1)	43 (51.8)	122 (55.7)
		To a moderate extent	13 (16.5)	10 (17.5)	8 (9.6)	31 (14.2)
		To a large extent	6 (7.6)	1 (1.8)	1 (1.2)	8 (3.7)
		Completely	–	–	–	–
20	I feel uncomfortable with the unpredictability of these patients	Not at all	26 (32.9)	14 (24.6)	26 (31.3)	66 (30.1)
		To a small extent	39 (49.4)	37 (64.9)	52 (62.7)	128 (58.4)
		To a moderate extent	13 (16.5)	6 (10.5)	3 (3.6)	22 (10)
		To a large extent	1 (1.3)	–	2 (2.4)	3 (1.4)
		Completely	–	–	–	–
21	I feel tense when interacting with these patients	Not at all	30 (38)	15 (26.3)	41 (49.4)	86 (39.3)
		To a small extent	43 (54.4)	40 (70.2)	40 (48.2)	123 (56.2)
		To a moderate extent	5 (6.3)	2 (3.5)	2 (2.4)	9 (4.1)
		To a large extent	1 (1.3)	–	–	1 (0.5)
		Completely	–	–	–	–
22	I feel irritated with the challenging behavior of these patients	Not at all	18 (22.8)	13 (22.8)	23 (27.7)	54 (24.7)
		To a small extent	46 (58.2)	40 (70.2)	54 (65.1)	140 (63.9)
		To a moderate extent	10 (12.7)	4 (7)	5 (6)	19 (8.7)
		To a large extent	4 (5.1)	–	1 (1.2)	5 (2.3)
		Completely	1 (1.3)	–	–	1 (0.5)
23	I feel angry with these patients’ lack of effort	Not at all	23 (29.1)	19 (33.3)	24 (28.9)	66 (30.1)
		To a small extent	41 (51.9)	31 (54.4)	52 (62.7)	124 (56.6)
		To a moderate extent	13 (16.5)	6 (10.5)	5 (6)	24 (11)
		To a large extent	2 (2.5)	1 (1.8)	2 (2.4)	5 (2.3)
		Completely	–	–	–	–
24	I feel contempt for the attitudes of these patients	Not at all	52 (65.8)	32 (56.1)	53 (63.9)	137 (62.6)
		To a small extent	23 (29.1)	24 (42.1)	27 (32.5)	74 (33.8)
		To a moderate extent	3 (3.8)	1 (1.8)	2 (2.4)	6 (2.7)
		To a large extent	1 (1.3)	–	1 (1.2)	2 (0.9)
		Completely	–	–	–	–
*Discrimination*						
25	I refer to these patients by their diagnosis or offense rather than their name	Not at all	69 (87.3)	42 (73.7)	71 (85.5)	182 (83.1)
		To a small extent	10 (12.7)	14 (24.6)	12 (14.5)	36 (16.4)
		To a moderate extent	–	1 (1.8)	–	1 (0.5)
		To a large extent	–	–	–	–
		Completely	–	–	–	–
26	I tend to speak to these patients in a simplified or condescending manner	Not at all	47 (59.5)	42 (73.7)	68 (81.9)	157 (71.7)
		To a small extent	22 (27.8)	15 (26.3)	14 (16.9)	51 (23.3)
		To a moderate extent	5 (6.3)	–	1 (1.2)	6 (2.7)
		To a large extent	5 (6.3)	–	–	5 (2.3)
		Completely	–	–	–	–
27	I remind these patients that I am the expert when they doubt my decisions	Not at all	32 (40.5)	33 (57.9)	51 (61.4)	116 (53)
		To a small extent	36 (45.6)	23 (40.4)	29 (34.9)	88 (40.2)
		To a moderate extent	6 (7.6)	–	2 (2.4)	8 (3.7)
		To a large extent	5 (6.3)	1 (1.8)	1 (1.2)	7 (3.2)
		Completely	–	–	–	–
28	I enable these patients to have a voice in decisions that affect them *	Not at all	23 (29.1)	6 (10.5)	9 (10.8)	38 (17.4)
		To a small extent	46 (58.2)	36 (63.2)	59 (71.1)	141 (64.4)
		To a moderate extent	7 (8.9)	9 (15.8)	12 (14.5)	28 (12.8)
		To a large extent	2 (2.5)	5 (8.8)	3 (3.6)	10 (4.6)
		Completely	1 (1.3)	1 (1.8)	–	2 (0.9)
29	I make an extra effort for these patients, regardless of their attitude or behavior *	Not at all	8 (10.1)	4 (7)	4 (4.8)	16 (7.3)
		To a small extent	43 (54.4)	30 (52.6)	48 (57.8)	121 (55.3)
		To a moderate extent	15 (19)	14 (24.6)	25 (30.1)	54 (24.7)
		To a large extent	10 (12.7)	9 (15.8)	5 (6)	24 (11)
		Completely	3 (3.8)	–	–	3 (1.4)
30	I am flexible with rules and restrictions for these patients when the situation allows it *	Not at all	7 (8.9)	3 (5.3)	4 (4.8)	14 (6.4)
		To a small extent	42 (53.2)	10 (17.5)	18 (21.7)	70 (32)
		To a moderate extent	17 (21.5)	20 (35.1)	28 (33.7)	65 (29.7)
		To a large extent	12 (15.2)	20 (35.1)	32 (38.6)	64 (29.2)
		Completely	1 (1.3)	4 (7)	1 (1.2)	6 (2.7)
31	I focus more on the containment than on the treatment of these patients	Not at all	28 (35.4)	10 (17.5)	15 (18.1)	53 (24.2)
		To a small extent	33 (41.8)	45 (78.9)	39 (47)	117 (53.4)
		To a moderate extent	15 (19)	1 (1.8)	22 (26.5)	38 (17.4)
		To a large extent	2 (2.5)	1 (1.8)	6 (7.2)	9 (4.1)
		Completely	–	–	–	–
32	I prioritize public safety over the rights of these patients	Not at all	16 (20.3)	6 (10.5)	8 (9.6)	30 (13.7)
		To a small extent	32 (40.5)	22 (38.6)	36 (43.4)	90 (41.1)
		To a moderate extent	25 (31.6)	12 (21.1)	27 (32.5)	64 (29.2)
		To a large extent	4 (5.1)	14 (24.6)	9 (10.8)	27 (12.3)
		Completely	1 (1.3)	3 (5.3)	1 (1.2)	5 (2.3)
33	I use coercive measures to correct these patients’ behavior	Not at all	38 (48.1)	19 (33.3)	48 (57.8)	105 (47.9)
		To a small extent	27 (34.2)	32 (56.1)	30 (36.1)	89 (40.6)
		To a moderate extent	10 (12.7)	6 (10.5)	4 (4.8)	20 (9.1)
		To a large extent	3 (3.8)	–	1 (1.2)	4 (1.8)
		Completely	–	–	–	–
34	I tend to dismiss the concerns or complaints of these patients when they seem exaggerated	Not at all	34 (43)	16 (28.1)	36 (43.4)	86 (39.3)
		To a small extent	32 (40.5)	37 (64.9)	43 (51.8)	112 (51.1)
		To a moderate extent	11 (13.9)	3 (5.3)	2 (2.4)	16 (7.3)
		To a large extent	1 (1.3)	1 (1.8)	2 (2.4)	4 (1.8)
		Completely	–	–	–	–
35	I avoid engaging with these patients whenever possible	Not at all	61 (77.2)	43 (75.4)	77 (92.8)	181 (82.6)
		To a small extent	14 (17.7)	14 (24.6)	6 (7.2)	34 (15.5)
		To a moderate extent	–	–	–	–
		To a large extent	1 (1.3)	–	–	1 (0.5)
		Completely	2 (2.5)	–	–	2 (0.9)
36	I tend to limit the responsibilities and tasks of these patients, even when they are capable	Not at all	53 (67.1)	31 (54.4)	61 (73.5)	145 (66.2)
		To a small extent	22 (27.8)	25 (43.9)	19 (22.9)	66 (30.1)
		To a moderate extent	3 (3.8)	1 (1.8)	2 (2.4)	6 (2.7)
		To a large extent	–	–	–	–
		Completely	–	–	1 (1.2)	1 (0.5)

a Includes all 36 items assessed during Rasch analyses; items 6, 12, 15, 16, 29 and 30 were removed from the final PROFS scale.

b The response options ‘to a very small extent’ and ‘to a small extent’ were merged into ‘to a small extent’.

c The response options ‘to a large extent’ and ‘to a very large extent’ were merged into ‘to a large extent’.

*Reverse scored items.

### Internal consistency

The final PROFS scale and its subscales showed acceptable to good Rasch-based reliability: total PROFS (PSI = 0.86; α = 0.90), Stereotypes (PSI = 0.82; α = 0.84), Prejudice (PSI = 0.75; α = 0.80), and Discrimination (PSI = 0.67; α = 0.78). Country−specific Cronbach’s alpha values for the total PROFS were 0.90 for Spain, 0.87 for the Netherlands, and 0.78 for Belgium, indicating adequate reliability across settings (**Supplementary Table 6**). Subscale alphas were adequate to high across countries for Stereotypes (α = 0.74–0.85) and Prejudice (α = 0.69–0.79), whereas reliability for Discrimination was acceptable in Spain (α = 0.77) but lower in the Netherlands (α = 0.68) and Belgium (α = 0.57), suggesting that this subscale functions less consistently in some countries.

Internal consistency of the external measures in the present sample was acceptable to good. Cronbach’s α for the AMTIO Negative Stereotypes subscale was 0.84, for the APDQ subscales 0.87 (S/V), 0.72 (A/R), and 0.70 (P/F), and for the SDS 0.89. For the IRI subscales, Cronbach’s a was 0.73. Cronbach’s a for the overall BIDR- 16 was 0.75, with values of 0.65 and 0.72 for the SDE and IM subscales, respectively.

### Construct validity

Spearman’s rank correlations between the PROFS subscales were moderate to strong (*ρ* = .39–.49, all *p* <.01), and each subscale correlated strongly with the PROFS total (*ρ* = .76–.81, all *p* <.01), indicating that the three PROFS components are related but distinguishable. Convergent validity was examined using Spearman’s rank correlations between the PROFS subscales and conceptually related external measures (**Table 4**). As hypothesized, Stereotypes showed a strong positive association with the ATMIO Negative Stereotypes (*ρ* = .58, *p* <.01), Prejudice correlated moderately to strongly with the APDQ subscales (*ρ* = .40–.55, all *p* <.01), and Discrimination demonstrated a moderate positive correlation with the SDS (*ρ* = .36, *p* <.01), indicating that higher levels of professional stigma on the PROFS were associated with higher levels of other stigma-related constructs.

**Table 4:** Convergent validity: Spearman’s rank correlations between PROFS subscales and external stigma-related measures (ATMIO, APDQ, SDS).

Measure	PROFS		
	Stereotypes	Prejudice	Discrimination
ATMIO NS	.58**	.53**	.53**
APDQ S/V	.35**	.55**	.52**
APDQ A/R	.35**	.40**	.43**
APDQ P/F	.40**	.40**	.43**
SDS	.47**	.33**	.36**

ATMIO NS, Negative Stereotypes subscale of the Attitudes towards Mentally Ill Offenders (ATMIO); APDQ S/V, Security/Vulnerability subscale of the Attitudes towards Personality Disorder Questionnaire (APDQ); APDQ A/R, Acceptance/Rejection subscale of the APDQ; APDQ P/F, Purpose/Futility subscale of the APDQ; SDS, Social Distance Scale.

** Significant at the p <.01 level (two-tailed).

Divergent validity was examined using Spearman’s rank correlations between the PROFS subscales and empathy and social desirability (**Table 5**). For empathy (IRI), Stereotypes and Discrimination correlated weakly and negatively with IRI Empathic Concern (*ρ* = −.24 and *ρ* = −.21, respectively, *p* <.01). Prejudice and Discrimination correlated weakly and negatively with IRI Perspective Taking (*ρ* = −.22 and *ρ* = −.21, respectively, *p* <.01), whereas the other correlations between PROFS subscales and IRI scores were non−significant. For social desirability (BIDR-16), Prejudice showed a weak negative correlation with the BIDR-16 Self-Deceptive Enhancement subscale (*ρ* = −.15, *p* <.05). All other correlations between PROFS subscales and the BIDR subscales were non-significant. Overall, these patterns suggest that the PROFS subscales are only minimally related to empathy and social desirability, supporting divergent validity.

**Table 5:** Divergent validity: Spearman’s rank correlations between PROFS subscales and empathy (IRI) and social desirability (BIDR-16).

Measure	PROFS		
	Stereotypes	Prejudice	Discrimination
IRI EC	-.24**	-.12	-.21**
IRI PT	-.11	-.22**	-.21**
BIDR-16 Total	.13	-.13	-.07
BIDR-16-SDE	.08	-.15*	.03
BIDR-16-IM	.11	-.09	-.12

IRI EC, Empathic Concern subscale of the Interpersonal Reactivity Index (IRI); IRI PT, Perspective Taking subscale of the IRI; BIDR-16, Balanced Inventory of Desirable Responding–Short Form; BIDR-16 SDE, Self-Deceptive Enhancement subscale of the BIDR-16; BIDR-16 IM, Impression Management subscale of the BIDR-16.

**Significant at the p <.01 level (two-tailed).

*Significant at the p <.05 level (two-tailed).

## Discussion

We evaluated the psychometric properties of the newly developed PROfessional Forensic Stigma scale (PROFS). The PROFS is designed to assess stigma towards forensic service users by capturing role- and context-specific manifestations of stigma among MHC professionals working with forensic service users. Its content validity was supported by a theory-driven item development process, complemented with expert consensus and input from users with lived experiences. Unlike many existing stigma measures, which are either not designed for forensic service users or do not focus on MHC professionals, the PROFS was developed to capture cognitive, emotional and behavioral manifestations of professional stigma in services providing MHC to this population.

The present study supports a multidimensional structure of the PROFS, comprising three conceptually coherent aspects: Stereotypes, Prejudice and Discrimination. This three-subscale solution was preferred over more fragmented, purely measurement-driven alternatives because it closely aligns with the underlying theoretical model of professional stigma and yields scores that are readily interpretable and useful for both research and clinical practice.

Across countries, median PROFS scores were generally low, and distributions showed pronounced floor effects, particularly for the Discrimination subscale, indicating that strongly expressed professional stigma was uncommon in this sample.

The PROFS total scale and its subscales demonstrated acceptable to good reliability. Rasch-based indices and internal consistency estimates supported stable measurement across countries, with the exception of the Discrimination subscale, which showed weaker reliability in the Netherlands and Belgium. Examination of country-specific “alpha if item deleted” statistics did not identify any single item whose removal would meaningfully improve internal consistency, suggesting that lower reliability here reflects overall subscale performance rather than the influence of a problematic item. The more heterogeneous professional composition of the Belgian sample, with a broader mix of roles and service settings, may have made some Discrimination items less applicable or differently salient for certain professional groups (75), which in turn can have reduced the internal consistency estimates. In addition, the targeting of Discrimination items at moderate to high stigma levels and the low variability in reported discriminatory behaviors likely limit the discriminative power of some items at lower stigma levels, thereby contributing to reduced internal consistency. This means that the Discrimination subscale may function less reliably in some contexts, implying that scores should be interpreted with more caution in these settings and that further refinement and cross-cultural validation in larger and more diverse samples are warranted.

Patterns of convergent and divergent correlations with external measures were largely consistent with hypotheses, supporting the construct validity of the three PROFS subscales. These findings suggest that the PROFS assesses stigmatizing beliefs, affective reactions and behavioral tendencies that are conceptually aligned with existing stigma instruments. At the same time, the PROFS showed only weak associations with broader interpersonal tendencies, consistent with the idea that it captures professional stigma rather than empathy or social desirability. Taken together, these findings provide initial support for the PROFS as a psychometrically sound measure of professional stigma towards forensic service users.

The dimensionality analyses further suggested that, while the Stereotypes and Discrimination subscales can be treated as essentially unidimensional, the Prejudice subscale may encompass minor subfacets (e.g. different affective reactions) rather than a perfectly homogeneous construct. Given the modest magnitude of this effect, we interpreted it as reflecting the complex and heterogeneous nature of prejudicial emotions in this context rather than as evidence against treating the Prejudice items as a single scale.

The Rasch person–item distributions showed that the PROFS items, and particularly the Discrimination subscale, are primarily targeted at moderate to high levels of professional stigma, indicating limited sensitivity at low stigma levels. Several items (14 in total) showed disordered thresholds, meaning that adjacent categories were not consistently distinguished. In line with Rasch guidelines, we interpreted this as evidence of suboptimal category functioning rather than fundamentally flawed items (76). Collapsing the original 7 response options into a 5-point format yielded slightly better fit and reliability while retaining more intuitive response options, and the current five-category format was therefore judged to offer the best balance between model fit and interpretability.

At the content level, these refinements also had implications for the breadth of stigma manifestations covered by the PROFS. Several items were removed, which inevitably reduced coverage of some content domains, particularly attributions of blame, stereotypical beliefs about dependency, prejudicial affective responses of pity and protectiveness, and behavioral tendencies such as making extra efforts and applying rules flexibly. Four of these items were positively worded, which can be cognitively more demanding when assessing a negative construct and may have contributed to their suboptimal psychometric performance (77).

In contrast, other items were retained despite psychometric limitations because they captured conceptually central aspects of professional stigma. Specifically, one Prejudice item addressing compassion was kept despite slight misfit, and two Discrimination items reflecting devaluation of the individual and avoidance were retained despite floor effects. These themes had been identified as core manifestations of stigma by forensic service users (9) and were therefore considered essential for content validity. Nonetheless, their observed psychometric limitations suggest that further refinement of these items may be warranted.

Similarly, a Discrimination item assessing prioritization of containment over therapeutic treatment showed small-to-moderate cross-national DIF and was retained. The observed DIF may reflect differences in service context rather than measurement bias, as Spanish and Dutch participants were more often employed in secure settings (i.e., forensic mental health hospitals or specialized forensic wards within prison) where containment and security are more salient features of everyday practice (78), whereas Belgian participants more often worked in community-based services. In this case, the cross-national DIF may thus reflect contextual differences, and item-level country comparisons should be interpreted with caution.

The PROFS was developed to capture professional, role-bound stigma in treatment contexts and contributes to stigma research by introducing a dedicated instrument for MHC professionals working with forensic populations. By operationalizing stereotypes (cognitive), prejudicial affective reactions (emotional), and discriminatory tendencies (behavioral) that are specific to these settings, the PROFS addresses aspects of stigma that are better aligned with the realities of working with forensic service users. Unlike measures of public stigma or attitudes toward people with mental illness, the PROFS is explicitly designed for MHC professionals in interaction with forensic service users, whose combination of mental illness and history of criminal offending raises distinct stigma concerns, and thus fills an important gap in available stigma measures for these settings. It appears well suited to monitor professional stigma and to inform the design of targeted anti-stigma interventions. It can support services in identifying and addressing stigma at team and organizational levels, thereby reducing the risk that negative attitudes become normalized and perpetuated in clinical decision-making (17, 79, 80).

In line with its three-subscale structure, we recommend that Stereotypes, Prejudice, and Discrimination scores are reported and interpreted primarily at the subscale level, with the overall PROFS score used as a summary index of general professional stigma rather than as a strongly validated single factor. The final 5-point response format (0 = not at all, 1 = to a small extent, 2 = to a moderate extent, 3 = to a large extent, 4 = completely) should be used and reported in future applications to ensure transparent score interpretation and comparability across studies. Although floor effects and some mistargeting, particularly within the Discrimination subscale, call for cautious interpretation of scores, the scale nonetheless offers a structured way to make otherwise implicit attitudes and practices visible and discussable in routine care. Its practical utility will ultimately depend on how these insights are integrated and translated into reflective supervision, team dialogue, or organizational learning processes that support a safe, constructive therapeutic environment for forensic service users and professionals.

This study had several limitations that should be acknowledged when interpreting these findings. First, there is a risk of selection bias. Participation was voluntary, recruitment occurred primarily via professional networks, and an over 80% completion criterion was applied, which likely favored relatively motivated and engaged professionals with more positive attitudes towards forensic service users. As a result, strongly stigmatizing attitudes may be under-represented, and the absolute stigma levels observed here may underestimate those in the wider field, particularly among non-responding professionals. In addition, the most extreme response category (‘completely’) was rarely endorsed, which may reflect genuinely low levels of strongly expressed stigma but also suggest that this label was perceived as too extreme, further contributing to floor effects and underuse of the upper end of the PROFS response scale. Second, all data were based on self-report, making the results susceptible to social desirability and role-related impression management. Although participation was anonymous and a general measure of socially desirable responding (BIDR-16) was included, this instrument does not capture profession-specific self-presentation concerns. Social desirability bias may therefore have led respondents to adjust their answers towards accepted or expected positions within a specific social and professional context (81), potentially resulting in under−reporting of explicitly prejudicial or discriminatory attitudes and thereby further contributing to the observed floor effects. As a consequence, the stigma levels reported here should be interpreted as conservative estimates of explicitly endorsed professional stigma. Third, the PROFS primarily captures professionals’ self-reported beliefs, emotions, and behavior, and thus relies on self-reflections about their own practice. It measures what professionals think they do but does not permit firm conclusions about actual behavior without complementary observational or behavioral data. Consequently, the present findings should be understood as reflecting explicit, self−acknowledged attitudes and behavioral tendencies rather than direct evidence of their behavior in everyday practice. Fourth, the sample showed an overrepresentation of female participants and psychologists. Stigma research among MHC professionals has yielded mixed findings regarding gender differences (23, 24, 35), and some studies suggest that psychologists may, on average, endorse less stigmatizing attitudes than other professional groups (82, 83). This may have contributed to lower overall stigma scores and limits generalizability to multidisciplinary teams in which males and other professional groups are more strongly represented. In addition, data collection relied on a non-probabilistic convenience sample from three countries, further limiting representativeness and generalizability across cultural, institutional, and professional contexts. Finally, the relatively small sample size may have constrained the precision of the item and scale parameter estimates and limits the stability of the Rasch and reliability results, particularly for more complex procedures such as DIF analyses across smaller subgroups (e.g. by country, age or gender), so that some aspects of item functioning and internal structure will need to be confirmed in larger, more diverse samples.

Future research should clarify whether the observed floor effects, mistargeting and disordered thresholds, particularly within the Discrimination subscale, are attributable to suboptimal item targeting, specific characteristics of the present sample, or the extreme endpoint label. Studies with larger and more representative samples of MHC professionals working with forensic service users across different service contexts are needed to evaluate this and, if necessary, to refine the wording of milder items to improve functioning at lower stigma levels. Because the final 30-item version of the PROFS was derived through item reduction and Rasch refinement within the present sample, studies in independent samples and other jurisdictions are needed to substantiate its psychometric properties and cross-cultural robustness. In addition, larger samples within each country and forensic system are required to examine the stability of the three-subscale structure, reliability and DIF patterns across different settings. To address limitations related to self-report and potential socially desirable responses, future studies could combine the PROFS with behavioral or vignette-based indicators (e.g., observed interactions, peer ratings, or responses to video- or vignette-based patient stimuli), as well as qualitative interviews, to obtain a more nuanced understanding of how professionals endorse and enact stigma in MHC settings. Such multi-method approaches would help to clarify potential under-reporting, test convergent validity with more direct indicators of stigmatizing behavior and capture more immediate emotional and behavioral reactions to concrete forensic patient profiles rather than the current generalized responses to forensic service users. This would also allow a more direct test of the attitude–behavior link assumed in stigma frameworks, in which stereotypes and prejudicial emotions are expected to shape discriminatory responses (1, 39, 84). Finally, longitudinal and intervention studies are needed to establish test–retest stability and temporal consistency, to evaluate sensitivity to change, and to examine the capacity of the PROFS to detect intervention effects on professional stigma over time.

## Conclusions

This study provides initial psychometric support for the PROFS as a multidimensional measure of professional stigma in groups of MHC professionals. While further validation in larger and more diverse samples is warranted, the PROFS can be used to identify stereotypes, prejudices and discriminatory tendencies that are salient in services providing mental healthcare to forensic service users.

## Data Availability

The datasets presented in this article are not readily available. The de‑identified data that support the findings of this study can be obtained from the corresponding author upon reasonable request. Because the sample is drawn from a limited number of mental health services, certain combinations of demographic and occupational variables could potentially enable re‑identification. Data sharing is therefore subject to ethical and legal restrictions and will require a data sharing agreement. Requests to access the datasets should be directed to ellen.vorstenbosch@sjd.es.
