# Supplementary material for: Psychometric evaluation of the professional forensic stigma scale
Source: Front Psychiatry. 2026 Jul 8;17:1806480. doi: 10.3389/fpsyt.2026.1806480 (PMC13390379; doi:10.3389/fpsyt.2026.1806480)
Supplement: Supplementary file 1 [file Supplementaryfile1.docx]

Supplementary Table S1. Item-level descriptive statistics of the PROFS original 7-point Likert scale (N=219)

|  |  | **N** | **Missing values** | **Mean (SD)** | **Median (IQR)** | **0** | **1** | **2** | **3** | **4** | **5** | **6** | **CITC** |
| --- | --- | --- | --- | --- | --- | --- | --- | --- | --- | --- | --- | --- | --- |
|  |  |  |  |  |  | **%** | **%** | **%** | **%** | **%** | **%** | **%** |  |
| 1 | Most of these patients can learn to control their impulses * | 219 | 0 | 2.59 (1.04) | 3 (1) | 3.2 | 9.6 | 32.4 | 37.0 | 15.5 | 2.3 | 0.0 | .359 |
| 2 | Most of these patients can make a positive contribution to society * | 219 | 0 | 2.54 (1.19) | 2 (1) | 5.0 | 11.4 | 33.8 | 29.7 | 14.2 | 5.9 | 0.0 | .443 |
| 3 | Most of these patients lack a sense of right and wrong | 219 | 0 | 2.00 (1.30) | 2 (2) | 11.4 | 28.8 | 25.6 | 19.2 | 12.3 | 2.7 | 0.0 | .288 |
| 4 | In general, these patients do not really want to change | 219 | 0 | 1.74 (1.13) | 2 (1) | 12.8 | 31.5 | 32 | 17.8 | 4.1 | 1.8 | 0.0 | .479 |
| 5 | These patients use their mental disorder to avoid responsibility | 219 | 0 | 1.63 (1.00) | 1 (1) | 17.4 | 35.6 | 23.3 | 15.5 | 6.8 | 1.4 | 0.0 | .562 |
| 6 | These patients are not to blame for their current situation * | 218 | 1 | 3.70 (1.31) | 4 (2) | 0.5 | 7.3 | 8.2 | 25.1 | 29.2 | 23.3 | 5.9 | .089 |
| 7 | In general, these patients lack the capacity to change | 219 | 0 | 2.16 (1.11) | 2 (2) | 7.3 | 19.2 | 37 | 25.1 | 10.0 | 1.4 | 0.0 | .421 |
| 8 | These patients manipulate to gain special treatment or privileges | 219 | 0 | 2.23 (1.35) | 2 (2) | 8.2 | 26.9 | 23.3 | 21.5 | 15.5 | 4.1 | 0.5 | .507 |
| 9 | These patients suddenly become aggressive, without any apparent cause | 219 | 0 | 1.36 (1.02) | 1 (1) | 16.4 | 50.2 | 18.3 | 11.4 | 3.2 | 0.5 | 0.0 | .505 |
| 10 | Most of these patients pose an inherent risk to others | 219 | 0 | 2.01 (1.31) | 2 (2) | 9.1 | 33.3 | 24.2 | 17.8 | 11.9 | 3.2 | 0.5 | .507 |
| 11 | Most of these patients will reoffend sooner or later | 218 | 1 | 2.30 (1.12) | 2 (1) | 3.2 | 20.5 | 36.1 | 26.5 | 9.6 | 3.2 | 0.5 | .448 |
| 12 | These patients will always depend on others | 219 | 0 | 2.54 (1.30) | 2 (1) | 5.9 | 14.2 | 30.6 | 25.6 | 17.8 | 4.6 | 1.4 | .304 |
| 13 | I feel compassion for these patients * | 219 | 0 | 2.77 (1.46) | 3 (2) | 5.0 | 13.7 | 25.6 | 29.7 | 11.0 | 11.0 | 4.1 | .252 |
| 14 | I feel optimistic about the future of these patients * | 219 | 0 | 2.94 (1.06) | 3 (2) | 1.4 | 6.8 | 23.3 | 40.2 | 22.8 | 4.6 | 0.9 | .452 |
| 15 | I feel protective of these patients * | 219 | 0 | 2.85 (1.29) | 3 (1) | 3.7 | 9.6 | 23.7 | 38.4 | 13.7 | 7.8 | 3.2 | .149 |
| 16 | I feel pity for these patients | 218 | 1 | 2.17 (1.47) | 2 (2) | 14.2 | 23.3 | 20.1 | 23.3 | 12.3 | 5.5 | 0.9 | -.033 |
| 17 | I feel indifferent towards the problems of these patients | 219 | 0 | 0.60 (0.88) | 0 (1) | 59.4 | 26.9 | 9.6 | 3.2 | 0.5 | 0.5 | 0.0 | .420 |
| 18 | I feel frustrated with the slow progress of these patients | 219 | 0 | 2.00 (1.47) | 2 (2) | 13.7 | 29.7 | 23.3 | 20.1 | 6.4 | 3.7 | 3.2 | .271 |
| 19 | I feel overwhelmed when treating these patients | 219 | 0 | 1.34 (1.15) | 1 (2) | 26.5 | 35.6 | 20.1 | 14.2 | 2.7 | 0.9 | 0.0 | .282 |
| 20 | I feel uncomfortable with the unpredictability of these patients | 219 | 0 | 1.11 (1.01) | 1 (2) | 30.1 | 42 | 16.4 | 10 | 0.9 | 0.5 | 0.0 | .425 |
| 21 | I feel tense when interacting with these patients | 219 | 0 | 0.85 (0.85) | 1 (1) | 39.3 | 41.6 | 14.6 | 4.1 | 0.5 | 0.0 | 0.0 | .441 |
| 22 | I feel irritated with the challenging behaviour of these patients | 219 | 0 | 1.21 (1.05) | 1 (1) | 24.7 | 44.7 | 19.2 | 8.7 | 1.8 | 0.5 | 0.5 | .501 |
| 23 | I feel angry with these patients' lack of effort | 219 | 0 | 1.14 (1.06) | 1 (2) | 30.1 | 41.6 | 15.1 | 11 | 1.8 | 0.5 | 0.0 | .541 |
| 24 | I feel contempt for the attitudes of these patients | 219 | 0 | 0.53 (0.81) | 0 (1) | 62.6 | 26.9 | 6.8 | 2.7 | 0.9 | 0.0 | 0.0 | .568 |
| 25 | I refer to these patients by their diagnosis or offense rather than their name | 219 | 0 | 0.21 (0.51) | 0 (0) | 83.1 | 13.2 | 3.2 | 0.5 | 0.0 | 0.0 | 0.0 | .349 |
| 26 | I tend to speak to these patients in a simplified or condescending manner | 219 | 0 | 0.47 (0.92) | 0 (1) | 71.7 | 17.8 | 5.5 | 2.7 | 1.8 | 0.5 | 0.0 | .388 |
| 27 | I remind these patients that I am the expert when they doubt my decisions | 219 | 0 | 0.73 (0.99) | 0 (1) | 53.0 | 31.5 | 8.7 | 3.7 | 3.2 | 0.0 | 0.0 | .419 |
| 28 | I enable these patients to have a voice in decisions that affect them * | 219 | 0 | 1.63 (1.18) | 2 (1) | 17.4 | 29.7 | 34.7 | 12.8 | 3.2 | 1.4 | 0.9 | .463 |
| 29 | I make an extra effort for these patients, regardless of their attitude or behaviour * | 218 | 1 | 2.25 (1.23) | 2 (1) | 7.3 | 16.9 | 38.4 | 24.7 | 6.8 | 4.1 | 1.4 | .212 |
| 30 | I am flexible with rules and restrictions for these patients when the situation allows it * | 219 | 0 | 2.79 (1.45) | 3 (2) | 6.4 | 15.1 | 16.9 | 29.7 | 21 | 8.2 | 2.7 | .245 |
| 31 | I focus more on the containment than on the treatment of these patients | 217 | 2 | 1.43 (1.17) | 1 (1) | 24.2 | 33.8 | 19.6 | 17.4 | 3.7 | 0.5 | 0 | .355 |
| 32 | I prioritize public safety over the rights of these patients | 216 | 3 | 2.16 (1.43) | 2 (2) | 13.7 | 22.4 | 18.7 | 29.2 | 10 | 2.3 | 2.3 | .312 |
| 33 | I use coercive measures to correct these patients' behaviour | 218 | 1 | 0.86 (1.06) | 1 (1) | 47.9 | 31.1 | 9.6 | 9.1 | 1.4 | 0.5 | 0.0 | .341 |
| 34 | I tend to dismiss the concerns or complaints of these patients when they seem exaggerated | 218 | 1 | 0.94 (1.01) | 1 (1) | 39.3 | 38.4 | 12.8 | 7.3 | 1.4 | 0.5 | 0.0 | .347 |
| 35 | I avoid engaging with these patients whenever possible | 218 | 1 | 0.25 (0.75) | 0 (0) | 82.6 | 13.2 | 2.3 | 0.0 | 0.5 | 0.0 | 0.9 | .282 |
| 36 | I tend to limit the responsibilities and tasks of these patients, even when they are capable | 218 | 1 | 0.46 (0.80) | 0 (1) | 66.2 | 25.6 | 4.6 | 2.7 | 0.0 | 0.0 | 0.5 | .329 |

SD: Standard deviation; IQR: Interquartile range; CITC: corrected item–total correlation.

Values in columns 0–6 are percentages of responses in each answer category.

* Reverse scored items

Supplementary Table S2. Rasch item statistics of the PROFS original 7-point Likert scale (N=219)

| Subscale /Item Nr. | | **Outfit MSQ** | **Infit MSQ** | **Location (logits)** | **SD of thresholds** | **Disordered thresholds ^a^** | **LID ^b^** | **DIF ^c^** |
| --- | --- | --- | --- | --- | --- | --- | --- | --- |
| *Stereotypes* | | | | | | | | |
| 1 | Most of these patients can learn to control their impulses * | 0.98 | 0.98 | 2.04 | 0.85 | **-** | i2 | - |
| 2 | Most of these patients can make a positive contribution to society * | 0.84 | 0.84 | 2.17 | 0.87 | **-** | - | - |
| 3 | Most of these patients lack a sense of right and wrong | 1.04 | 1.01 | 2.67 | 0.90 | **-** | - | - |
| 4 | In general, these patients do not really want to change | 0.72 | 0.73 | 2.80 | 1.03 | Y | - | - |
| 5 | These patients use their mental disorder to avoid responsibility | 0.82 | 0.82 | 2.86 | 0.88 | **-** | - | - |
| 6 | These patients are not to blame for their current situation * | **1.90** | **1.77** | -0.87 | 1.58 | Y | - | - |
| 7 | In general, these patients lack the capacity to change | 0.88 | 0.85 | 2.54 | 0.97 | **-** | - | - |
| 8 | These patients manipulate to gain special treatment or privileges | 0.81 | 0.82 | 0.41 | 0.96 | **-** | - | - |
| 9 | These patients suddenly become aggressive, without any apparent cause | 0.68 | 0.71 | 2.97 | 1.33 | Y | - | - |
| 10 | Most of these patients pose an inherent risk to others | 0.80 | 0.76 | 0.47 | 1.19 | **-** | - | - |
| 11 | Most of these patients will reoffend sooner or later | 0.83 | 0.82 | 0.22 | 1.37 | **-** | - | - |
| 12 | These patients will always depend on others | 1.02 | 1.01 | 0.09 | 0.83 | **-** | - | - |
| *Prejudice* | | | | | | | | |
| 13 | I feel compassion for these patients * | **1.21** | 1.17 | -0.74 | 0.58 | Y | **-** | - |
| 14 | I feel optimistic about the future of these patients * | 0.85 | 0.85 | -0.73 | 0.70 | **-** | **-** | - |
| 15 | I feel protective of these patients * | 1.17 | 1.15 | -0.76 | 0.41 | Y | **-** | - |
| 16 | I feel pity for these patients | **1.73** | **1.61** | -0.23 | 0.40 | **-** | **-** | - |
| 17 | I feel indifferent towards the problems of these patients | 0.85 | 0.91 | 2.47 | 0.34 | Y | **-** | - |
| 18 | I feel frustrated with the slow progress of these patients | 0.98 | 0.93 | -0.42 | 0.67 | Y | **-** | - |
| 19 | I feel overwhelmed when treating these patients | 0.97 | 0.87 | 2.05 | 0.56 | Y | i20 | - |
| 20 | I feel uncomfortable with the unpredictability of these patients | 0.67 | 0.70 | 2.21 | 0.74 | Y | i21 | - |
| 21 | I feel tense when interacting with these patients | 0.71 | 0.75 | 2.37 | 0.86 | Y | i22 | - |
| 22 | I feel irritated with the challenging behaviour of these patients | 0.61 | 0.61 | 0.15 | 0.94 | Y | **-** | - |
| 23 | I feel angry with these patients' lack of effort | 0.69 | 0.71 | 2.18 | 0.74 | Y | **-** | - |
| 24 | I feel contempt for the attitudes of these patients | 0.55 | 0.66 | 2.50 | 0.34 | Y | **-** | - |
| *Discrimination* | | | | | | | | |
| 25 | I refer to these patients by their diagnosis or offense rather than their name | 0.62 | 0.86 | 2.34 | 0.34 | Y | - | - |
| 26 | I tend to speak to these patients in a simplified or condescending manner | 1.03 | 0.86 | 2.16 | 0.20 | Y | - | - |
| 27 | I remind these patients that I am the expert when they doubt my decisions | 0.83 | 0.86 | 1.95 | 0.49 | Y | - | - |
| 28 | I enable these patients to have a voice in decisions that affect them * | 0.80 | 0.79 | -0.74 | 0.99 | Y | - | - |
| 29 | I make an extra effort for these patients, regardless of their attitude or behaviour * | 1.04 | 1.01 | -1.06 | 0.93 | Y | - | - |
| 30 | I am flexible with rules and restrictions for these patients when the situation allows it * | 1.13 | 1.13 | -1.29 | 0.49 | Y | - | - |
| 31 | I focus more on the containment than on the treatment of these patients | 0.94 | 0.92 | 1.29 | 0.57 | - | - | - |
| 32 | I prioritize public safety over the rights of these patients | 1.15 | 1.06 | -1.02 | 0.43 | Y | - | - |
| 33 | I use coercive measures to correct these patients' behaviour | 0.97 | 0.90 | 1.87 | 0.55 | Y | - | - |
| 34 | I tend to dismiss the concerns or complaints of these patients when they seem exaggerated | 0.79 | 0.79 | 1.77 | 0.66 | Y | - | - |
| 35 | I avoid engaging with these patients whenever possible | 0.68 | 0.78 | -0.27 | 6.41 | Y | - | - |
| 36 | I tend to limit the responsibilities and tasks of these patients, even when they are capable | 1.00 | 0.81 | -0.22 | 0.58 | Y | - | - |

^a^ Disordered response thresholds (Y) versus ordered thresholds (–); thresholds were considered ordered when their locations increased monotonically across categories.

^b^ Local item dependence (LID) was examined via correlations among standardized residuals; item pairs with residual correlations ≥ 0.30 above the average residual correlation are flagged as indicating potential LID.

^c^ Significant differential item functioning for gender (g), age group (a), and/or country (c). Only effects with Bonferroni‑adjusted p < .001 are flagged.

* Reverse scored items

MSQ values indicating misfit are shown in bold.

Supplementary Table S3. Item-level descriptive statistics for the PROFS with collapsed answer categories (N=219)

|  |  | **N** | **Missing values** | **Median (IQR)** | **0** | **1** | **2** | **3** | **4** | **CITC** |
| --- | --- | --- | --- | --- | --- | --- | --- | --- | --- | --- |
|  |  |  |  |  | **%** | **%** | **%** | **%** | **%** |  |
| *Stereotypes* | | | | | | | | | | |
| 1 | Most of these patients can learn to control their impulses * | 219 | 0 | 2 (1) | 3.2 | 42.0 | 37.0 | 17.8 | 0.0 | .351 |
| 2 | Most of these patients can make a positive contribution to society * | 219 | 0 | 1 (1) | 5.0 | 45.2 | 29.7 | 20.1 | 0.0 | .391 |
| 3 | Most of these patients lack a sense of right and wrong | 219 | 0 | 1 (1) | 11.4 | 54.3 | 19.2 | 15.1 | 0.0 | .313 |
| 4 | In general, these patients do not really want to change | 219 | 0 | 1 (0) | 12.8 | 63.5 | 17.8 | 5.9 | 0.0 | .485 |
| 5 | These patients use their mental disorder to avoid responsibility | 219 | 0 | 1 (0) | 17.4 | 58.9 | 15.5 | 8.2 | 0.0 | .556 |
| 6 | These patients are not to blame for their current situation * | 218 | 1 | 3 (1) | 0.5 | 15.6 | 25.2 | 52.8 | 5.9 | -.006 |
| 7 | In general, these patients lack the capacity to change | 219 | 0 | 1 (1) | 7.3 | 56.2 | 25.1 | 11.4 | 0.0 | .392 |
| 8 | These patients manipulate to gain special treatment or privileges | 219 | 0 | 1 (1) | 8.2 | 50.2 | 21.5 | 19.6 | 0.5 | .474 |
| 9 | These patients suddenly become aggressive, without any apparent cause | 219 | 0 | 1 (0) | 16.4 | 68.5 | 11.4 | 3.7 | 0.0 | .465 |
| 10 | Most of these patients pose an inherent risk to others | 219 | 0 | 1 (1) | 9.1 | 57.5 | 17.8 | 15.1 | 0.5 | .462 |
| 11 | Most of these patients will reoffend sooner or later | 218 | 1 | 1 (1) | 3.2 | 56.9 | 26.6 | 12.8 | 0.5 | .431 |
| 12 | These patients will always depend on others | 219 | 0 | 1 (1) | 5.9 | 44.7 | 25.6 | 22.4 | 1.4 | .266 |
| *Prejudice* | | | | | | | | | | |
| 13 | I feel compassion for these patients * | 219 | 0 | 2 (2) | 5.0 | 39.3 | 29.7 | 21.9 | 4.1 | .212 |
| 14 | I feel optimistic about the future of these patients * | 219 | 0 | 2 (2) | 1.4 | 30.1 | 40.2 | 27.4 | 0.9 | .428 |
| 15 | I feel protective of these patients * | 219 | 0 | 2 (1) | 3.7 | 33.3 | 38.4 | 21.5 | 3.2 | .183 |
| 16 | I feel pity for these patients | 218 | 1 | 1 (1) | 14.2 | 43.6 | 23.4 | 17.9 | 0.9 | -.005 |
| 17 | I feel indifferent towards the problems of these patients | 219 | 0 | 0 (1) | 59.4 | 36.5 | 3.2 | 0.9 | 0.0 | .430 |
| 18 | I feel frustrated with the slow progress of these patients | 219 | 0 | 1 (1) | 13.7 | 53.0 | 20.1 | 10.0 | 3.2 | .250 |
| 19 | I feel overwhelmed when treating these patients | 219 | 0 | 1 (1) | 26.5 | 55.7 | 14.2 | 3.7 | 0.0 | .288 |
| 20 | I feel uncomfortable with the unpredictability of these patients | 219 | 0 | 1 (1) | 30.1 | 58.4 | 10.0 | 1.4 | 0.0 | .353 |
| 21 | I feel tense when interacting with these patients | 219 | 0 | 1 (1) | 39.3 | 56.2 | 4.1 | 0.5 | 0.0 | .387 |
| 22 | I feel irritated with the challenging behaviour of these patients | 219 | 0 | 1 (0) | 24.7 | 63.9 | 8.7 | 2.3 | 0.5 | .461 |
| 23 | I feel angry with these patients' lack of effort | 219 | 0 | 1 (1) | 30.1 | 56.6 | 11.0 | 2.3 | 0.0 | .495 |
| 24 | I feel contempt for the attitudes of these patients | 219 | 0 | 0 (1) | 62.6 | 33.8 | 2.7 | 0.9 | 0.0 | .549 |
| *Discrimination* | | | | | | | | | | |
| 25 | I refer to these patients by their diagnosis or offense rather than their name | 219 | 0 | 0 (0) | 83.1 | 16.4 | 0.5 | 0.0 | 0.0 | .315 |
| 26 | I tend to speak to these patients in a simplified or condescending manner | 219 | 0 | 0 (1) | 71.7 | 23.3 | 2.7 | 2.3 | 0.0 | .405 |
| 27 | I remind these patients that I am the expert when they doubt my decisions | 219 | 0 | 0 (1) | 53.0 | 40.2 | 3.7 | 3.2 | 0.0 | .396 |
| 28 | I enable these patients to have a voice in decisions that affect them * | 219 | 0 | 1 (0) | 17.4 | 64.4 | 12.8 | 4.6 | 0.9 | .382 |
| 29 | I make an extra effort for these patients, regardless of their attitude or behaviour * | 218 | 1 | 1 (1) | 7.3 | 55.5 | 24.8 | 11.0 | 1.4 | .173 |
| 30 | I am flexible with rules and restrictions for these patients when the situation allows it * | 219 | 0 | 2 (2) | 6.4 | 32.0 | 29.7 | 29.2 | 2.7 | .225 |
| 31 | I focus more on the containment than on the treatment of these patients | 217 | 2 | 1 (0) | 24.2 | 53.9 | 17.5 | 4.1 | 0.0 | .346 |
| 32 | I prioritize public safety over the rights of these patients | 216 | 3 | 1 (1) | 13.7 | 41.7 | 29.6 | 12.5 | 2.3 | .289 |
| 33 | I use coercive measures to correct these patients' behaviour | 218 | 1 | 1 (1) | 47.9 | 40.8 | 9.2 | 1.8 | 0.0 | .340 |
| 34 | I tend to dismiss the concerns or complaints of these patients when they seem exaggerated | 218 | 1 | 1 (1) | 39.3 | 51.4 | 7.3 | 1.8 | 0.0 | .328 |
| 35 | I avoid engaging with these patients whenever possible | 218 | 1 | 0 (0) | 82.6 | 15.6 | 0.0 | 0.5 | 0.9 | .327 |
| 36 | I tend to limit the responsibilities and tasks of these patients, even when they are capable | 218 | 1 | 0 (1) | 66.2 | 30.3 | 2.8 | 0.0 | 0.5 | .298 |

IQR: Interquartile range; CITC: corrected item–total correlation.

Values in columns 0–6 are percentages of responses in each answer category.

* Reverse scored items

Supplementary Table S4. Rasch item statistics of the PROFS scale with collapsed answer categories (N=219)

| Subscale /Item Nr. | | **Outfit MSQ** | **Infit MSQ** | **Location (logits)** | **SD thresholds** | **Disordered thresholds ^a^** | **LID ^b^** | **DIF ^c^** |
| --- | --- | --- | --- | --- | --- | --- | --- | --- |
| *Stereotypes* | | | | | | | | |
| 1 | Most of these patients can learn to control their impulses * | 1.04 | 0.99 | 3.05 | 2.24 | **-** | **-** | - |
| 2 | Most of these patients can make a positive contribution to society * | 0.85 | 0.86 | 3.23 | 1.94 | **-** | **-** | - |
| 3 | Most of these patients lack a sense of right and wrong | 1.01 | 0.99 | -1.50 | 1.69 | Y | **-** | - |
| 4 | In general, these patients do not really want to change | 0.73 | 0.75 | 4.02 | 2.06 | **-** | **-** | - |
| 5 | These patients use their mental disorder to avoid responsibility | 0.81 | 0.80 | 4.29 | 1.69 | Y | **-** | - |
| 6 | These patients are not to blame for their current situation * | **1.78** | **1.58** | 4.38 | 2.22 | **-** | **-** | - |
| 7 | In general, these patients lack the capacity to change | 0.97 | 0.91 | 3.78 | 2.07 | **-** | **-** | - |
| 8 | These patients manipulate to gain special treatment or privileges | 0.84 | 0.85 | 0.32 | 1.71 | Y | **-** | - |
| 9 | These patients suddenly become aggressive, without any apparent cause | 0.69 | 0.73 | 4.50 | 2.10 | Y | **-** | - |
| 10 | Most of these patients pose an inherent risk to others | 0.82 | 0.77 | 0.40 | 1.91 | Y | **-** | - |
| 11 | Most of these patients will reoffend sooner or later | 0.81 | 0.82 | 0.08 | 2.56 | **-** | **-** | - |
| 12 | These patients will always depend on others | 1.11 | 1.07 | -0.11 | 1.80 | **-** | **-** | - |
| *Prejudice* | | | | | | | | |
| 13 | I feel compassion for these patients * | **1.25** | 1.19 | -1.07 | 1.63 | **-** | i15 | - |
| 14 | I feel optimistic about the future of these patients * | 0.90 | 0.90 | -1.07 | 2.16 | **-** | - | - |
| 15 | I feel protective of these patients * | 1.19 | 1.16 | -1.10 | 1.75 | **-** | - | - |
| 16 | I feel pity for these patients | **1.63** | **1.50** | -0.32 | 1.16 | Y | - | - |
| 17 | I feel indifferent towards the problems of these patients | 0.82 | 0.88 | 4.09 | 1.18 | Y | - | - |
| 18 | I feel frustrated with the slow progress of these patients | 0.97 | 0.93 | -0.62 | 1.51 | Y | - | - |
| 19 | I feel overwhelmed when treating these patients | 0.88 | 0.84 | 3.31 | 1.49 | **-** | i20 | - |
| 20 | I feel uncomfortable with the unpredictability of these patients | 0.76 | 0.77 | 3.57 | 1.78 | **-** | i21 | - |
| 21 | I feel tense when interacting with these patients | 0.76 | 0.78 | 3.88 | 1.91 | **-** | - | - |
| 22 | I feel irritated with the challenging behaviour of these patients | 0.62 | 0.62 | 0.24 | 1.80 | Y | - | - |
| 23 | I feel angry with these patients' lack of effort | 0.75 | 0.75 | 3.50 | 1.58 | **-** | - | - |
| 24 | I feel contempt for the attitudes of these patients | 0.60 | 0.67 | 4.14 | 1.12 | Y | - | - |
| *Discrimination* | | | | | | | | |
| 25 | I refer to these patients by their diagnosis or offense rather than their name | 0.71 | 0.90 | 3.50 | 6.31 | Y | - | - |
| 26 | I tend to speak to these patients in a simplified or condescending manner | 0.92 | 0.85 | 3.30 | 0.89 | Y | - | - |
| 27 | I remind these patients that I am the expert when they doubt my decisions | 0.85 | 0.86 | 3.01 | 1.30 | Y | - | - |
| 28 | I enable these patients to have a voice in decisions that affect them * | 0.84 | 0.81 | -0.93 | 1.84 | Y | - | - |
| 29 | I make an extra effort for these patients, regardless of their attitude or behaviour * | 1.10 | 1.04 | -1.36 | 2.00 | **-** | - | - |
| 30 | I am flexible with rules and restrictions for these patients when the situation allows it * | 1.17 | 1.16 | -1.72 | 1.33 | **-** | - | - |
| 31 | I focus more on the containment than on the treatment of these patients | 0.93 | 0.90 | 2.14 | 1.54 | **-** | - | - |
| 32 | I prioritize public safety over the rights of these patients | 1.10 | 1.04 | -1.34 | 1.39 | **-** | - | - |
| 33 | I use coercive measures to correct these patients' behaviour | 0.93 | 0.89 | 2.89 | 1.09 | **-** | - | - |
| 34 | I tend to dismiss the concerns or complaints of these patients when they seem exaggerated | 0.81 | 0.81 | 2.74 | 1.41 | Y | - | - |
| 35 | I avoid engaging with these patients whenever possible | 0.62 | 0.76 | -0.29 | 12.88 | Y | - | - |
| 36 | I tend to limit the responsibilities and tasks of these patients, even when they are capable | 0.89 | 0.84 | -0.22 | 7.68 | Y | - | - |

^a^ Disordered response thresholds (Y) versus ordered thresholds (–); thresholds were considered ordered when their locations increased monotonically across categories.

^b^ Local item dependence (LID) was examined via correlations among standardized residuals; item pairs with residual correlations ≥ 0.30 above the average residual correlation are flagged as indicating potential LID.

^c^ Significant differential item functioning for gender (g), age group (a), and/or country (c). Only effects with Bonferroni‑adjusted p < .001 are flagged.

* Reverse scored items

MSQ values indicating misfit are shown in bold.

Supplementary Table S5. Rasch item statistics of the final PROFS scale (N=219)

| Subscale /Item Nr. | | **Outfit MSQ** | **Infit MSQ** | **Location (logits)** | **SD thresholds** | **Disordered thresholds ^a^** | **LID ^b^** | **DIF ^c^** |
| --- | --- | --- | --- | --- | --- | --- | --- | --- |
| *Stereotypes* | | | | | | | | |
| 1 | Most of these patients can learn to control their impulses * | 1.14 | 1.07 | 2.55 | 2.53 | **-** | - | - |
| 2 | Most of these patients can make a positive contribution to society * | 0.95 | 0.96 | 2.87 | 2.19 | **-** | - | - |
| 3 | Most of these patients lack a sense of right and wrong | 1.05 | 1.05 | 3.78 | 1.90 | Y | - | - |
| 4 | In general, these patients do not really want to change | 0.72 | 0.76 | 4.70 | 2.28 | **-** | - | - |
| 5 | These patients use their mental disorder to avoid responsibility | 0.82 | 0.80 | 4.83 | 1.88 | **-** | - | - |
| 7 | In general, these patients lack the capacity to change | 1.02 | 0.99 | 3.39 | 2.32 | **-** | - | - |
| 8 | These patients manipulate to gain special treatment or privileges | 0.94 | 0.96 | -0.10 | 1.92 | Y | - | - |
| 9 | These patients suddenly become aggressive, without any apparent cause | 0.75 | 0.80 | 5.74 | 2.31 | Y | - | - |
| 10 | Most of these patients pose an inherent risk to others | 0.90 | 0.85 | 0.00 | 2.12 | Y | - | - |
| 11 | Most of these patients will reoffend sooner or later | 0.86 | 0.87 | -0.35 | 2.85 | **-** | - | - |
| *Prejudice* | | | | | | | | |
| 13 | I feel compassion for these patients * | **1.40** | **1.31** | -1.49 | 1.86 | **-** | - | - |
| 14 | I feel optimistic about the future of these patients * | 1.09 | 1.07 | -1.53 | 2.43 | **-** | - | - |
| 17 | I feel indifferent towards the problems of these patients | 0.92 | 0.96 | 4.24 | 1.36 | Y | - | - |
| 18 | I feel frustrated with the slow progress of these patients | 1.07 | 1.03 | -0.95 | 1.70 | **-** | - | - |
| 19 | I feel overwhelmed when treating these patients | 1.03 | 0.91 | 3.15 | 1.70 | **-** | - | - |
| 20 | I feel uncomfortable with the unpredictability of these patients | 0.80 | 0.81 | 3.52 | 2.02 | **-** | - | - |
| 21 | I feel tense when interacting with these patients | 0.75 | 0.78 | 3.96 | 2.14 | **-** | - | - |
| 22 | I feel irritated with the challenging behaviour of these patients | 0.63 | 0.63 | 0.05 | 2.00 | Y | - | - |
| 23 | I feel angry with these patients' lack of effort | 0.79 | 0.78 | 3.42 | 1.80 | **-** | - | - |
| 24 | I feel contempt for the attitudes of these patients | 0.62 | 0.69 | 4.32 | 1.28 | Y | - | - |
| *Discrimination* | | | | | | | | |
| 25 | I refer to these patients by their diagnosis or offense rather than their name | 1.11 | 1.02 | 3.37 | 7.33 | Y | - | - |
| 26 | I tend to speak to these patients in a simplified or condescending manner | 0.65 | 0.89 | 3.13 | 0.92 | Y | - | - |
| 27 | I remind these patients that I am the expert when they doubt my decisions | 0.90 | 0.86 | 2.71 | 1.37 | Y | - | - |
| 28 | I enable these patients to have a voice in decisions that affect them * | 0.90 | 0.86 | -1.49 | 2.06 | Y | - | - |
| 31 | I focus more on the containment than on the treatment of these patients | 0.96 | 0.95 | 1.57 | 1.73 | - | - | c |
| 32 | I prioritize public safety over the rights of these patients | 1.09 | 1.08 | -1.95 | 1.65 | - | - | - |
| 33 | I use coercive measures to correct these patients' behaviour | 0.95 | 0.87 | 2.53 | 1.23 | - | - | - |
| 34 | I tend to dismiss the concerns or complaints of these patients when they seem exaggerated | 0.76 | 0.79 | 2.31 | 1.55 | Y | - | - |
| 35 | I avoid engaging with these patients whenever possible | 0.51 | 0.70 | -0.76 | 14.65 | Y | - | - |
| 36 | I tend to limit the responsibilities and tasks of these patients, even when they are capable | 0.81 | 0.79 | -0.70 | 8.60 | Y | - | - |

^a^ Disordered response thresholds (Y) versus ordered thresholds (–); thresholds were considered ordered when their locations increased monotonically across categories.

^b^ Local item dependence (LID) was examined via correlations among standardized residuals; item pairs with residual correlations ≥ 0.30 above the average residual correlation are flagged as indicating potential LID.

^c^ Significant differential item functioning for gender (g), age group (a), and/or country (c). Only effects with Bonferroni‑adjusted p < .001 are flagged.

* Reverse scored items

MSQ values indicating misfit are shown in bold.

Figure S1-3. Person–item maps for each of the PROFS subscales

| 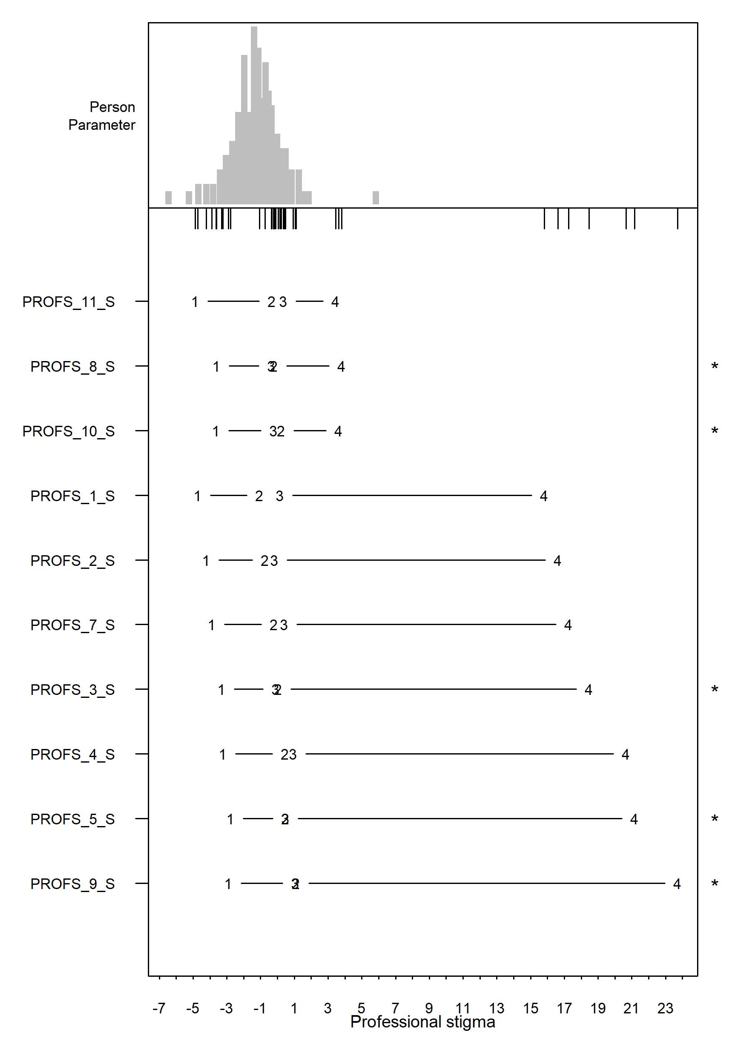   1. Stereotypes | 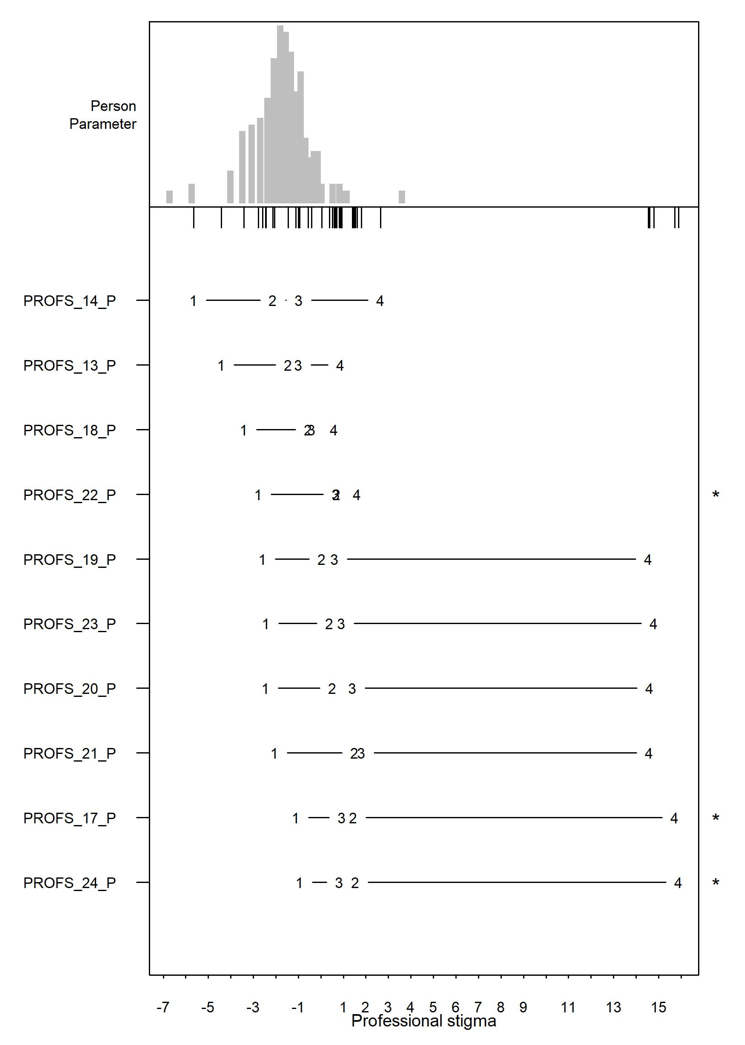   1. Prejudice |
| --- | --- |
| 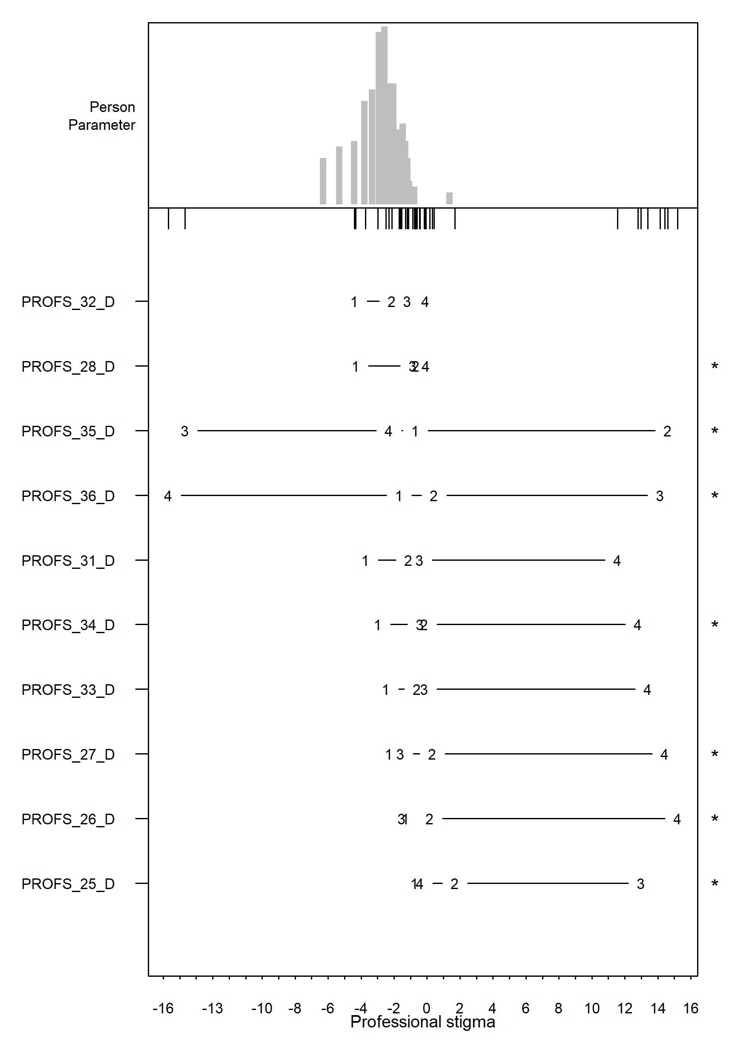   1. Discrimination |  |

Table S6. Cronbach's alpha values for internal consistency of the PROFS and its subscales, per country and total sample (N=219)

|  | **Spain** | **The Netherlands** | **Belgium** | **Total** |
| --- | --- | --- | --- | --- |
| **PROFS Total** | .90 | .87 | .78 | **.90** |
| **PROFS Stereotypes** | .84 | .85 | .74 | **.84** |
| **PROFS Prejudice** | .79 | .75 | .69 | **.80** |
| **PROFS Discrimination** | .77 | .68 | .57 | **.78** |
